# Suppressors of *ipl1-2* in Components of a Glc7 Phosphatase Complex, Cdc48 AAA ATPase, TORC1, and the Kinetochore

**DOI:** 10.1534/g3.112.003814

**Published:** 2012-12-01

**Authors:** Lucy C. Robinson, Joshua Phillips, Lina Brou, Evan P. Boswell, Kelly Tatchell

**Affiliations:** Department of Biochemistry and Molecular Biology, Louisiana State University Health Sciences Center, Shreveport, Louisiana 71130

**Keywords:** Aurora B, *IPl1*, kinetochore, *NDC80*, *GLC7*

## Abstract

Ipl1/Aurora B is the catalytic subunit of a protein kinase complex required for chromosome segregation and nuclear division. Before anaphase, Ipl1 is required to establish proper kinetochore-microtubule associations and to regulate the spindle assembly checkpoint (SAC). The phosphatase Glc7/PP1 opposes Ipl1 for these activities. To investigate Ipl1 and Glc7 regulation in more detail, we isolated and characterized mutations in the yeast *Saccharomyces cerevisiae* that raise the restrictive temperature of the *ipl-2* mutant. These suppressors include three intragenic, second-site revertants in *IPL1*; 17 mutations in Glc7 phosphatase components (*GLC7*, *SDS22*, *YPI1*); two mutations in *SHP1*, encoding a regulator of the AAA ATPase Cdc48; and a mutation in *TCO89*, encoding a subunit of the TOR Complex 1. Two revertants contain missense mutations in microtubule binding components of the kinetochore. *rev76* contains the missense mutation *duo1-S115F*, which alters an essential component of the DAM1/DASH complex. The mutant is cold sensitive and arrests in G2/M due to activation of the SAC. *rev8* contains the missense mutation *ndc80-K204E*. K204 of Ndc80 corresponds to K166 of human Ndc80 and the human Ndc80 K166E variant was previously shown to be defective for microtubule binding *in vitro*. In a wild-type *IPL1* background, *ndc80-K204E* cells grow slowly and the SAC is activated. The slow growth and cell cycle delay of *ndc80-K204E* cells are partially alleviated by the *ipl1-2* mutation. These data provide biological confirmation of a biochemically based model for the effect of phosphorylation on Ndc80 function.

The protein kinase Aurora B (Ipl1 in *Saccharomyces cerevisiae*, Ark1 in *Schizosaccharomyces pombe*, *Air-2* in *Caenorhabditis elegans*) plays a pivotal role in ensuring bipolar attachment of chromosomes to the mitotic spindle. As the catalytic subunit of the tetrameric chromosome passenger complex, it associates with chromosomes early in mitosis and then accumulates at kinetochores, where it phosphorylates components of the kinetochore before anaphase (Ruchaud *et al.* 2007). After anaphase, Aurora B accumulates at the spindle midzone, where it has additional substrates involved in cytokinesis. The kinetochore is a large protein complex consisting of the inner kinetochore complex, which makes direct contact with centromeric chromatin, the outer kinetochore, which contains microtubule binding proteins that track the minus ends of growing and shrinking kinetochore microtubules, and a central domain that tethers the inner and outer kinetochore complexes [reviewed in (Santaguida and Musacchio 2009)]. Aurora B phosphorylates outer kinetochore proteins to regulate microtubule-binding dynamics, which is required to establish a bipolar arrangement of chromosomes on the mitotic spindle. Aurora B activity also is required for the spindle assembly checkpoint (SAC), which delays anaphase until all chromosomes are under bipolar attachment.

Tension at kinetochores, brought about by bipolar association of condensin-tethered chromosomes in the mitotic spindle, originally was proposed by Nicklas and Koch (Nicklas and Koch 1969) to regulate kinetochore microtubule dynamics. It is now thought that kinetochore-microtubule tension directly regulates the phosphorylation state of Aurora B kinetochore substrates. An attractive model for the coupling of tension to kinetochore substrate phosphorylation suggests that tension pulls the outer kinetochore away from the inner kinetochore (Andrews *et al.* 2004; Liu *et al.* 2009; Maresca and Salmon 2009; Uchida *et al.* 2009; Vanoosthuyse and Hardwick 2009). Indeed, a gradient of Aurora B activity is centered on inner centromeres of mammalian cells. Reduced Aurora B kinase activity at kinetochores under tension, in combination with a possible increase in protein phosphatase activity, leads to reduced phosphorylation and less dynamic kinetochore microtubule binding, and silencing of the SAC [reviewed by (Lampson and Cheeseman 2011)].

Multiple lines of evidence indicate that type 1 protein phosphatase (PP1 in mammals, Glc7 in *S. cerevisiae*, Dis2 and Sds21 in *S. pombe*, and *Ceglc-7* in *C. elegans*) is largely responsible for the dephosphorylation of Aurora B substrates during mitosis. Loss of function mutations in PP1 genes induce mitotic arrest in *S. pombe* (Ishii *et al.* 1996), *S. cerevisiae* (Hisamoto *et al.* 1994; MacKelvie *et al.* 1995), *Aspergillus nidulans* (Doonan and Morris 1989), and *Drosophila* (Axton *et al.* 1990), and anti-PP1 antibodies induce mitotic arrest when injected into mammalian cells (Fernandez *et al.* 1992). PP1 mutations in *S. cerevisiae* suppress the temperature sensitivity of *ipl1* mutants (Francisco *et al.* 1994; Hsu *et al.* 2000) and the phenotype of *air-2(RNAi)* can be suppressed by decreasing PP1 activity [*Ceglc-7(RNAi)*] in *C. elegans* (Hsu *et al.* 2000). In mammalian cells, PP1 localizes to kinetochores during mitosis (Trinkle-Mulcahy *et al.* 2003) and inhibition of PP1 activity suppresses defects associated with reduced Aurora B activity (Emanuele *et al.* 2008; Wang *et al.* 2008). The mitotic arrest of some *glc7* mutants requires the SAC (Bloecher and Tatchell 1999; Sassoon *et al.* 1999), but PP1 is also required for SAC silencing (Pinsky *et al.* 2009; Vanoosthuyse and Hardwick 2009). Together, these results are consistent with the idea that PP1 acts on Aurora B substrates to regulate kinetochore microtubule dynamics and SAC silencing.

PP1 activity is regulated by a large number of regulatory/targeting subunits that direct PP1 catalytic activity toward specific substrate(s) [reviewed by (Virshup and Shenolikar 2009; Bollen *et al.* 2010)]. A degenerate motif, the so-called RVxF motif found on many targeting subunits, is an essential interaction motif required for PP1c binding and regulation (Egloff *et al.* 1997). The conserved outer kinetochore protein Spc105 (KNL1 in mammals, Spc7 in *S. pombe*) contains both an RVxF motif and a less frequent SILK motif found in a subset of PP1 binding proteins (Hendrickx *et al.* 2009). A *KNL1* mutant whose product cannot bind PP1 (*KNL1^RVSF/AAAA^*) is lethal, and PP1 is not found at kinetochores in *KNL1^RVSF/AAAA^* cells (Liu *et al.* 2010). The mutant has enhanced Aurora B-dependent phosphorylation at the outer kinetochore and destabilized kinetochore microtubule attachments (Liu *et al.* 2010). Mutants in *KNL1* orthologs in *S. pombe* (*spc7*) and *S. cerevisiae* (*spc105*) that cannot bind PP1 also are inviable due to activation of the SAC (Meadows *et al.* 2011; Rosenberg *et al.* 2011). Tethering PP1 directly to an Spc105 variant that cannot bind PP1 (Spc105^RVSF-RASA^) rescues cell lethality but, in contrast, tethering PP1 to wild-type Spc105 is lethal and cannot be rescued by disruption of the SAC (Rosenberg *et al.* 2011). These results suggest that the level of PP1 targeted to the outer kinetochore is under exquisite control. Serine residues in both PP1 binding motifs in KNL1 (RV**S**F and **S**ILK) are phosphorylated by Aurora B *in vitro* and *in vivo* in human cells (Welburn *et al.* 2010). Phosphomimetic variants in KNL1 reduce microtubule and PP1 binding (Welburn *et al.* 2010). These findings suggest that KNL1 participates in a feed forward circuit regulating Aurora B substrate phosphorylation.

PP1 also binds to the kinesin-8 family members Klp5-Klp6 in *S. pombe*, where PP1 binding is thought to contribute to SAC silencing (Meadows *et al.* 2011). In *S. cerevisiae*, PP1 binds to the kinetochore/spindle protein Fin1, which assists in targeting PP1 to the kinetochore (Akiyoshi *et al.* 2009b). Misregulation of Fin1 results in premature silencing of the SAC in a PP1-dependent manner. However, Fin1 is not essential, suggesting that Fin1 activities overlap with those of other kinetochore proteins.

In addition to KNL1/Spc105/Spc7, Klp5-Klp6, and Fin1, several other PP1 regulators have been implicated with roles in opposing Ipl1/Aurora B activity at the kinetochore in *S. cerevisiae*. Mutations in *GLC8* (Tung *et al.* 1995), *SDS22* (Peggie *et al.* 2002), *YPI1* (Bharucha *et al.* 2008), and *SHP1* (Cheng and Chen 2010) suppress the temperature sensitivity of *ipl1-2* or *ipl1-321* mutations, implying that their products regulate PP1 activity opposing Ipl1. Although these proteins are evolutionarily conserved, they are not integral components of kinetochores and their precise roles are poorly understood. Sds22 and Ypi1 can form a ternary complex with Glc7 (Hazbun *et al.* 2003; Pedelini *et al.* 2007) and inhibit phosphatase activity *in vitro* (Garcia-Gimeno *et al.* 2003; Lesage *et al.* 2007; Pedelini *et al.* 2007) although both behave genetically as positive regulators of PP1 activity. Sds22 in fission yeast has been shown to change the specificity of PP1 activity, increasing PP1 activity toward histone H3 and reducing its activity toward phosphorylase a (Stone *et al.* 1993). In vertebrate cells, Sds22 has been proposed to have a more canonical targeting role. It associates with PP1 at kinetochores and appears to regulate the PP1 activity opposing Aurora B (Posch *et al.* 2010). Interestingly, an essential histone H2A variant from *Tetrahymena thermophila* that is necessary to dephosphorylate histone 3 serine 10 at the end of mitosis contains an Sds22-related domain that binds PP1 (Song *et al.* 2012). However, both Sds22 and Ypi1 have been reported to have additional biological activities. Sds22 in *Drosophila* has been implicated in regulating the actin cytoskeleton to control epithelial cell architecture (Grusche *et al.* 2009; Shao *et al.* 2010; Jiang *et al.* 2011) and mitotic exit (Kunda *et al.* 2012), and Ypi1 regulates ion homeostasis in *S. cerevisiae* independently of Glc7 (Marquina *et al.* 2012).

Because the assay for suppression of *ipl1* temperature sensitivity has only been used to test mutants previously known to influence Glc7 activity, it is not known which additional gene products might act in Ipl1-dependent processes. We therefore conducted a classical genetic screen for suppressors of *ipl1-2*. In addition to new alleles of *GLC7*, *SHP1*, *YPI1*, and *SHP1*, the screen uncovered dominant alleles of *IPL1*, a mutant in the TOR complex 1 (TORC1) pathway, and mutants in *DUO1* and *NDC80*, two components of the outer kinetochore.

## Materials and Methods

### Yeast strains and media

The yeast strains used in this study are listed in Supporting Information, Table S1 and are congenic to KT1112 [*MAT***a** *leu2ura3his3* (Stuart *et al.* 1994)] and KT1113 (Frederick and Tatchell 1996). The *ipl1-2* mutation (Chan and Botstein 1993) was introduced into the KT1112 background by seven serial backcrosses. Yeast strains were transformed by the method of Gietz *et al.* (Gietz *et al.* 1992). Growth of yeast was in 1% yeast extract, 2% Bacto peptone, and 2% glucose (Yeast Extract Peptone Dextrose, *i.e.*, YPD) medium or in synthetic complete (SC) medium (Sherman *et al.* 1986), or modifications of these media as stated. Tetrad analysis was performed as described previously (Rose *et al.* 1990). Culture temperatures are indicated. 13myc-tagged alleles of *PDS1* and *IPL1* were generated using the method of Longtine *et al.* (Longtine *et al.* 1998) with F2 and R1 primers listed in Table S2. Polymerase chain reaction (PCR) products with the tagged alleles were introduced into the wild-type diploid strain KT1112/KT1113 by transformation; G418-resistant transformants were sporulated and protein extracts from G418-resistant meiotic progeny were tested for the presence of the tagged protein by immunoblot.

### Bacterial growth and DNA manipulation

All plasmids were amplified in *Escherichia coli* strain DH5α. DH5α was grown in LB medium supplemented with ampicillin to select for plasmids. Transformation of chemically competent bacterial cells was as described (Fritsch *et al.* 1989). Restriction enzymes (Promega), DNA ligase (NEB), and high-fidelity DNA polymerase for PCR (Bio-Rad and Phenix) were used according to the manufacturer’s instructions. Yeast genomic DNA was isolated using the YeaStar kit (Zymo Research), and yeast plasmid DNA was isolated using the Zymoprep kit (Zymo Research). Plasmids were purified from *E. coli* using the Zyppy miniprep kit (Zymo Research) or the GenElute mimiprep kit (Sigma-Aldrich). DNA restriction fragments were purified from agarose gel slices using the ZymoClean kit (Zymo Research).

### Mutant isolation

Strain KT1963 (*ipl1-2*) was grown overnight at 24°. Then, 250-μL aliquots of a 1/100 dilution were plated onto YPD plates and uncovered plates were irradiated with 5000 μJ/CM^2^ of ultraviolet (UV) light in a UVP-CL1000 UV crosslinker. After incubation in the dark at 24° for 24 hr, the plates were incubated for 3 d at 33°. Revertant colonies were tested for growth on YPD medium at temperatures ranging from 14° to 37° and on YPD medium containing 3% formamide, 0.1 M LiCl, 0.1 M CsCl, or 5 mM caffeine. Revertants with one or more growth defects and those that grew at 37° were backcrossed to an *ipl1-2* strain, and tetrad analysis was performed to confirm that *ipl1* suppression was due to a single Mendelian mutation and to test whether any growth defects were linked to suppression. Mutants were tested for complementation by strains carrying known *ipl1* suppressor mutations or for genetic linkage to known *ipl1* suppressor mutations. Tentative assignments were confirmed by DNA sequence analysis of the indicated suppressor locus from the mutant. In each case, the mutant locus was amplified by PCR and the DNA sequence of the ORF was obtained using the primers listed in Table S2. DNA sequences of PCR products and plasmids were determined by Macrogen USA (Rockville, MD). DNA sequences were compared with database sequences at the Saccharomyces Genome Database web site using the BLAST algorithm (Altschul *et al.* 1990).

### Cloning of the suppressor loci

*IPL1* meiotic segregants of KT2878 (rev8), KT2961 (rev76), and KT2967 (rev81) were transformed with a yeast genomic library in the *CEN URA3* vector YCp50 (a generous gift from Doug S. Conklin), and transformants were selected by growth on SC-URA medium at 24°. Transformants were replica plated onto YPD medium containing 3% formamide and incubated for 2−5 d at 30°. Colonies that grew on formamide medium were transferred to SC-URA and retested for resistance to formamide. Positive transformants were grown in nonselective conditions (YPD), transferred to SC+5-FOA medium to select for plasmid loss, and retested for growth on formamide medium. Plasmid DNA was isolated from transformants whose formamide resistance required the *URA3* plasmid, amplified in *E. coli*, and used to transform the original yeast strains. DNA sequences obtained from the genomic inserts of plasmids that conferred formamide resistance were compared to the Saccharomyces Genome Database using BLAST to identify the chromosome segment contained within each plasmid.

To ensure that the *ndc80* and *duo1* alleles are the suppressor mutations in rev8 and 76, respectively, we generated clones of these alleles and introduced these into diploid strains heterozygous for both *ipl1-2* and deletion of one of the two genes. The diploid transformants were sporulated and tetrads dissected. The progeny of diploid transformants with vector alone showed two viable spore clones per tetrad, and roughly 1/2 of these were temperature-sensitive for growth. None of the viable spore clones was resistant to G418. In contrast, progeny of *ndc80Δ*/+ *ipl1-2*/+ transformants with pRS316:*ndc80-8* include more than 2 viable spore clones per tetrad. We recovered 14 G418-resistant spore clones from 22 tetrads. All of these carry the plasmid marker, and those that have *ipl1-2* grow at 30°. This confirms that the *ndc80-8* allele is responsible for suppression of the *ipl1-2* inability to grow at 30°. We observed similar results for *ipl1-2*/+ *duo1Δ*/+ diploid transformants with pRS303:*duo1-76* (the *duo1-76* fragment contains ARS717, so pRS303:*duo1-76* is a replicating plasmid). In this case, we recovered 13 G418-resistant clones from 24 tetrads. All carry the plasmid marker and all grow at 30°. Figure S3 shows drop growth tests for four clones each of the *ipl1-2* and *ipl1-2 duo1-76* and *ipl1-2 ndc80-8* genotypes.

To generate pRS316:*ndc80-8* and pRS303:*duo1-76*, we used PCR with high-fidelity polymerase to amplify the genomic loci from strain KT3257 (*ndc80-8*) and strain KT3386 (*duo1-76*). Phenix HiFi polymerase was used to amplify *ndc80-8*, and HotStar (QIAGEN) was used to amplify *duo1-76*. Both polymerases were used according to manufacturer’s instructions. Genomic DNA was prepared using the YeaStar kit (Zymo Research). PCR primers were DUO1-Fa and DUO1-Ra, and NDC80-Fa and NDC80-Ra (Table S2). The NDC80 Fa and Ra primers contain *Bam*1H and *Xba*I sites, respectively. The *ndc80-8* PCR product was purified using the Clean and Concentrate kit (Zymo Research), then digested with *Bam*1H and *Xba*I. The digestion product was again purified using the Clean and Concentrate kit, and was ligated to *Bam*1H and *Xba*I-digested pRS316. The *duo1-76* PCR product was cloned via its A overhangs using the pGEM-T Easy kit (Promega). A *Sac*II-Sal fragment containing *duo1-76* was then cloned into pRS303. For both mutant alleles, the entire sequences of the cloned PCR products were determined to ensure the presence of the appropriate mutations and the absence of undesired changes.

### Microscopy

We imaged Glc7-mCitrine and Sds22-mCitrine in live cells and Ypi1-13Myc by indirect immunofluorescence as described (Bharucha *et al.* 2008). The Myc-tagged *YPI1* allele was chosen over an mCitrine-tagged allele due to a reduced activity of the mCitrine-tagged variant (Bharucha *et al.* 2008).

### Immunoblot analysis

Total protein extracts were prepared from yeast cultures grown to log phase using glass bead lysis in TCA (Davis *et al.* 1993). Protein extracts were electrophoresed through 4–20% gradient gels (Criterion; Bio-Rad), gels were blotted to nitrocellulose filters (Protran BA83; Whatman), and filters were probed with indicated primary antibodies followed by horseradish peroxidase-conjugated secondary antibodies (Bio-Rad). The anti-GFP antibody is an affinity-purified rabbit antibody generously provided by J. Nathan Davis, LSUHSC-S. HA-tagged and myc-tagged proteins were detected using the 12CA5 and 9E10 mouse monoclonal antibodies, respectively. The antiphosphoglycerate kinase antibody used as a loading reference is a mouse monoclonal antibody to yeast Pgk1 (Molecular Probes). Antibody binding was detected using the Immobilon ECL reagents (Millipore) and the Chemidoc system (Bio-Rad). Quantity One software (Bio-Rad) was used to image and quantitate signal on immunoblots. Blots were stripped before reprobing with reference antibody using Western Reprobe (G Biosciences).

## Results and Discussion

The *ipl1-2* mutant was originally identified by Chan and Botstein (Chan and Botstein 1993) in a screen for mutants with increased ploidy. Ipl1 activity in this mutant, as measured by the phosphorylation levels of histone H3Ser10, is reduced at 24° but growth rate is not affected at 24° (Hsu *et al.* 2000). However, *ipl1-2* cells lose chromosomes and viability rapidly at 30° (Tatchell *et al.* 2011). To survey in an unbiased way for mutations that suppress the temperature sensitivity of *ipl1-2*, we mutagenized an *ipl1-2* strain (KT1963) with UV light and isolated revertants that grew at 33° (see *Materials and Methods* for details). In most cases, we focused on revertants in which *ipl1-2* suppression segregated as a single Mendelian allele and cosegregated with a growth trait such as sensitivity to low temperature or to formamide, caffeine, or divalent cations in the growth medium. After placing suppressors into complementation groups, we showed that most are genetically linked to a predicted gene. In some cases, we cloned the loci by complementation of an associated growth defect. Finally, sequence analysis was performed on each mutant allele to identify the lesion responsible for suppression. Results for the 25 mutations that we identified as suppressors of *ipl1-2* are summarized in Table 1.

**Table 1:** Summary of *ipl1-2* suppressor mutations

Suppressor Locus Mutant Allele	Revertant No.	Mutation (S)	Residue in Human Ortholog
*IPL1*			
* IPL1-R150K H352Y*	14,21	G^449^A, C^1054^T	R139 H340
* IPL1-S167L H352Y*	16	C^500^T, C^1054^T	M136 H340
* IPL1-G347E H352Y*	20	G^1040^A, C^1054^T	K335 H340
*GLC7* (Type 1 protein phosphatase [PP1] catalytic subunit)			
* glc7-L15S*	106	T^44^C	L16
* glc7-L37F*	85	A^111^T, T^175^A	L38
* glc7-L71S*	82	T^737^C	L72
* glc7-L74P*	39, 62, 89	T^746^C	L75
* glc7-Y92N R141K*	55	T^799^A, G^947^A	Y93 R142
* glc7-S99L*	84	T^820^C, C^821^T	S100
* glc7-K112E*	107	A^859^G	K113
* glc7-F118S*	102	T^878^C	F119
* glc7-Y136N*	73	T^931^A	Y137
* glc7-Q293D*	112	A^1403^C	Q294
*YPI1* (Glc7/PP1 regulatory subunit)			
* ypi1-F74S*	52	T^221^C	Y64
* ypi1-F74L*	95	T^222^A	Y64
*SDS22* (Glc7/PP1 regulatory subunit)			
* sds22-F177S*	47	T^530^C	I204
* sds22-W187R*	53	T^559^A	F214
* sds22-D2N D119N*	74	G^4^A, G^355^A	D148
* sds22-77 (C-terminal frameshift)**^a^*	77	ΔC^1014^	
* sds22-E163I L329P*	94	^486^AGA^488^GAT, T^984^C	E192 V350
*SHP1* (subunit of Cdc48/p97 AAA AATPase)			
* shp1-FS99**^b^*	99	A insertion after 368, T^370^C	
* shp1-FS105**^c^*	105	A insertion after 873, G^877^A	
*TCO89* (nonessential subunit of TOR Complex 1)			
* tco89-71*	71	four missense codons and a stop codon*^d^*	not conserved
*DUO1* (subunit of DAM/DASH complex)			
* duo1-S115F*	76, 81	C^344^T	not conserved
*NDC80* (subunit of Ndc80 complex)			
* ndc80-K204E*	8	A^610^G	K166

a Frameshift at final codon (G 338 underlined) resulting in stop after 22 missense codons: …YIRG* →…YIRGDLDQEMIFFLYTHTNIYIYIYI.*

b Frameshift after codon 122 (underlined) resulting in stop after 15 missense codons: …GSGNN… →…GSGNKPQVYELFGYGKRSS*

c Frameshift after codon 291 (underlined) resulting in stop after 2 missense codons: …NVYKKLD… →…NVYKKIK*

d Tatchell *et al.* 2011.

### Intragenic *IPL1* revertants

**Figure 1:**
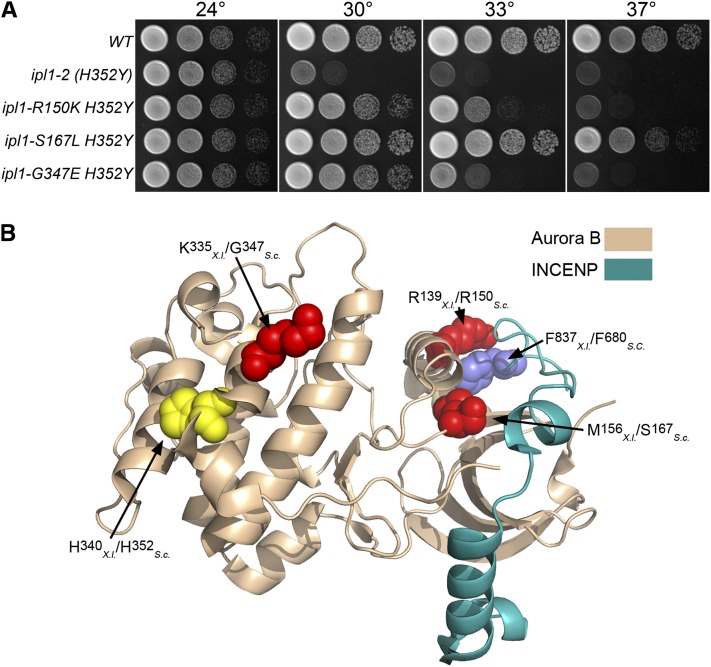
Intragenic *ipl1-2* suppressors. (A) Cultures of *WT* (KT1112), *ipl1-2 (H352Y)* (KT1829), *ipl1-2 R150K H352Y* (KT2865), *ipl1-S167L H352Y* (KT2867), and *ipl1-G347E H352Y* (KT2869) strains were serially diluted onto YPD medium and imaged after 40 hr at the designated temperatures. (B) The locations of intragenic *ipl1-2* suppressor mutations are mapped on the *X. laevis* Aurora B- INCENP structure [2BFX.pdb (Sessa *et al.* 2005)]. The highlighted amino acid residues are represented as space-filling models. The location of the *ipl1-2* mutation H352Y is shown in yellow. Residues altered by intragenic suppressor mutations are red, and the amino acid residue in INCENP that associates with R139 is blue. Note that both S167 and R150 are predicted to lie near the interface with INCENP/Sli15.

We recovered several strong and dominant suppressors, as shown by the ability of diploids between these revertants and *ipl1-2* strains to grow at 33−37°. Spore clones with the *ipl1-2* phenotype were never recovered from crosses between these revertants and a wild-type strain, indicating that the suppressor loci are tightly linked to *IPL1*. The sequence of the *IPL1* locus from two such suppressor mutants is identical to that of the wild-type *IPL1* locus, indicating that these two are true revertants. Sequence analysis of the other intragenic suppressor mutants revealed that these contain the original *ipl1-2* missense mutation (H352Y) and an additional missense mutation: R150K, S167L or G347E. *IPI1-S167L H352Y* strains grow nearly as well as wild-type strains at 33°, *IPI1-R150K H352Y* mutants have an intermediate growth phenotype, whereas *IPI1-G347E H352Y* mutants grow poorly at 33° (Figure 1A).

The C-terminal domain of Sli15, a positive regulator of Ipl1 (Kim *et al.* 1999), binds to and activates Ipl1 *in vitro* (Kang *et al.* 2001). Crystal structure of the Sli15-Ipl1 orthologous pair from *Xenopus laevis* (INCENP-Aurora B) reveals that R150 (R139 in Aurora B from *X. laevis*) lies at the Aurora B/INCENP interface and is part of a pocket on the surface of Aurora B that directly associates with the highly conserved residue F837 in INCENP [Figure 1B (Sessa *et al.* 2005)]. This pocket lies near the invariant E141 residue that is required for ATP binding and catalysis. It is likely that INCENP binding to Aurora B through this pocket allows for the proper orientation of E141. Interestingly, mutation of the adjacent residue R151K in Ipl1 results in reduced binding to Sli15 and reduced kinase activity (Kotwaliwale *et al.* 2007). Ipl1 S167 (M156 in *X. laevis* Aurora B) also lies close to the Aurora B/INCENP interface. One explanation for the suppressive effects of these mutations is that they increase the affinity of Ipl1 for Sli15, thereby effectively increasing the activity of the Ipl1-2 protein. G347 is in the protein kinase subdomain XI (Hanks and Hunter 1995), which is involved in stabilizing the large C-terminal lobe of the kinase. The substitution lies close to the *ipl1-2* mutation (H352Y), but this glycine residue is not highly conserved in Aurora B proteins from metazoans.

### Extragenic *ipl1-2* suppressors in the *GLC7* pathway

Most of the suppressor loci are unlinked to *ipl1-2*, as shown by the ability to recover strains with the original *ipl1-2* phenotype from crosses between revertant and wild-type strains. We identified twenty-two different suppressor mutations in seven genes. The largest group of extragenic suppressors contains mutations altering Glc7 phosphatase components that were previously reported as *ipl1* suppressor loci. We identified ten new alleles of *GLC7*, five *SDS22* alleles, two *YPI1* alleles, and two *SHP1* alleles.

#### GLC7:

**Figure 2:**
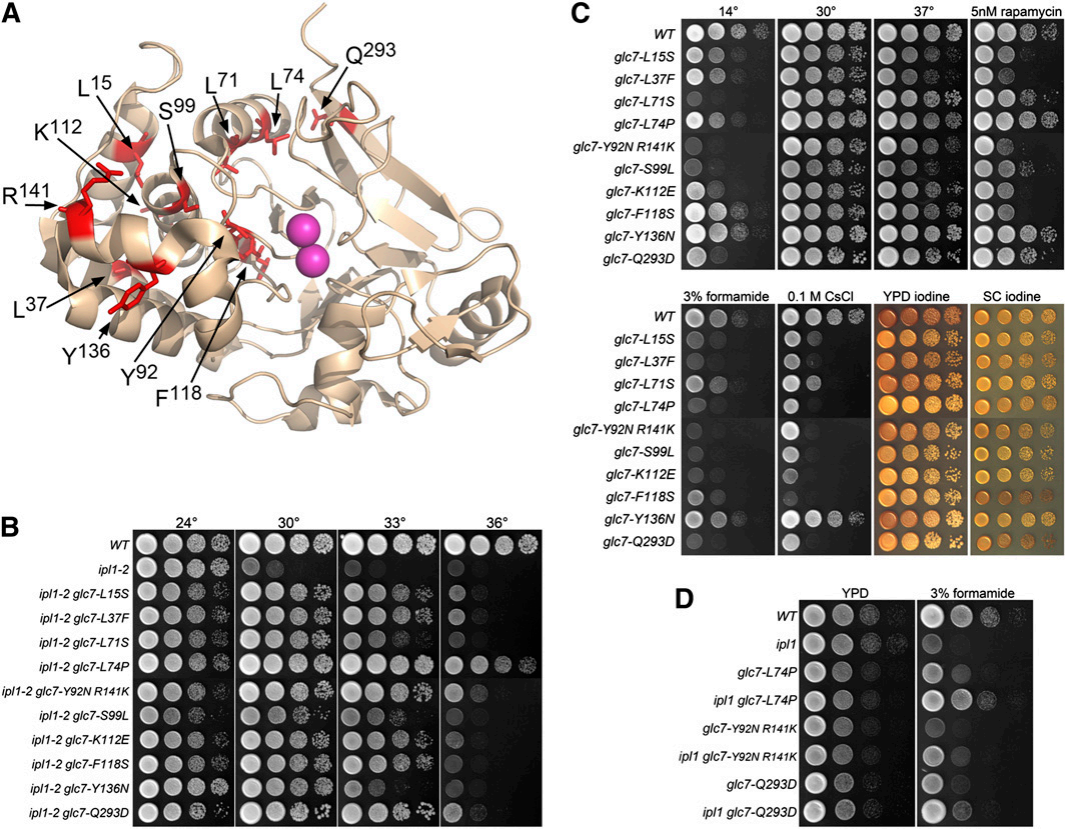
Extragenic *ipl1-2* suppressors in *GLC7*. (A) Locations of *GLC7 ipl1-2* suppressor changes mapped on the structure of rabbit PP1α [1FJM.pdb (Goldberg *et al.* 1995)]. The amino acid residues altered in the *ipl1* suppressor mutants are stick representations in red. The two Mn^2+^ ions in the active site are in magenta. (B) Cultures of *WT* (KT1113), *ipl1-2* (KT1829), *ipl1-2 glc7-L15S* (KT3062), *ipl1-2 glc7-L37F* (KT2940), *ipl1-2 glc7-L71S* (KT2938), *ipl1-2 glc7-L74P* (KT3361), *ipl1-2 glc7-Y92N R141K* (KT3365), *ipl1-2 glc7-S99L* (KT2939), *ipl1-2 glc7-K112E* (KT3064), *ipl1-2 glc7-F118S* (KT3059), *ipl1-2 glc7-Y136N* (KT3358), and *ipl1-2 glc7-Q293D* (KT3066) strains were serially diluted onto YPD medium and imaged after 40 hr at the designated temperatures. (C) Cultures of WT (KT1113), *glc7-L15S* (KT3304), *glc7-L37F* (KT2973), *glc7-L71S* (KT2969), *glc7-L74P* (KT3359), *glc7-Y92N R141K* (KT3363), *glc7-S99L* (KT2970), *glc7-K112E* (KT3308), *glc7-F118S* (KT3302), *glc7-Y136N* (KT3355), and *glc7-Q293D* (KT3310) strains were serially diluted onto the designated medium and imaged at the designated temperatures. All media, with the exception of SC, are either YPD or YPD supplemented with the indicated components. The YPD 14° plates were incubated for 14 d. All other plates were incubated for 40 hr. YPD iodine and SC iodine panels indicate cells grown on YPD or SC media for 40 hr and then stained with iodine vapor. (D) Cultures of WT (KT1113), *ipl1-2* (KT1829), *glc7-L74P* (KT3359), *ipl1-2 glc7-L74P* (KT3361), *glc7-Y92N R141K* (KT3363), *ipl1-2 glc7-Y92N R141K* (KT3365), *glc7-Q293D* (KT3310), and *ipl1-2 glc7-Q293D* (KT3066) strains were serially diluted onto YPD medium and YPD medium containing 3% formamide and imaged after 24 and 72hr, respectively, at 24°.

The 10 new *GLC7* alleles are all missense mutations that alter residues identical to those in mammalian PP1 orthologs. One allele, Y92N R141K, is a double mutant. L74P was isolated three times, but each other allele was recovered once. We have highlighted the mutated residues on the crystal structure of rabbit PP1 shown in Figure 2A. Most mutated residues are in the N-terminal lobe of PP1, and a majority of these are buried in the interior of the protein. The surface residues altered in *ipl1-2* suppressor strains are K112, Y137, R141, and Q293. It is worth noting that many *glc7* mutations previously reported to suppress *ipl1* mutants also are located in the N-terminal lobe of PP1 {*glc7-10* [F135L] (Andrews and Stark 2000); *glc7-127* [K110A, K112A], and *glc7-129* [D137A and E138A] (Baker *et al.* 1997)}, although the significance is as yet unclear.

Our new *glc7* mutants are quite variable in their ability to raise the restrictive temperature of *ipl1-2*. As shown in Figure 2B, *glc7-Y136N* only weakly suppresses *ipl1-2* at 33°, whereas *glc7-L74P* allows the *ipl1-2* mutant to grow weakly at 36°. We also compared the phenotypes of our collection of *glc7* mutants in a wild-type *IPL1* background. Growth defects common to many of the mutants include cold sensitivity and sensitivity to formamide, CsCl, rapamycin, and caffeine (Figure 2C). However, each mutant strain has a unique phenotype, and no trait correlates perfectly with the ability to suppress *ipl1-2*. These phenotypes are due to the *glc7* alleles rather than an accompanying mutation because each trait cosegregates with *ipl1* suppression in backcrosses. We believe that the highly individual phenotypes reflect the large number of Glc7 binding proteins that regulate substrate specificity.

Although our preliminary screen indicated that the *glc7* suppressor mutations are recessive for suppression, spot tests of diploid strains heterozygous for the *glc7* mutant and homozygous for *ipl1-2* revealed that all are partially dominant, as shown by some growth at 30° and 33° (Figure S1A). This finding is in contrast to suppressor mutants in *SDS22*, *YPI1*, and *SHP1*, which are completely recessive (Figure S1, B−D). One possibility is that the Glc7 variants sequester other cofactors required for phosphatase function, such as Sds22 or Ypi1. In this regard, it is worth noting that *SDS22* was originally identified in *S. pombe* and *S. cerevisiae* as a dosage suppressor of *dis2-11* (Ohkura and Yanagida 1991), *glc7-12* (MacKelvie *et al.* 1995), and *glc7-Y170* (Hisamoto *et al.* 1995).

If Glc7 directly opposes Ipl1 to dephosphorylate substrates involved in chromosome segregation, then we would expect that some traits associated with the *GLC7* mutations would be more severe in an *IPL1* background than in the *ipl1-2* background. As shown in Figure 2D, several *glc7* mutants containing the *ipl-2* allele grow better on YPD medium containing 3% formamide than either the *ipl1-2* or *glc7* single mutants. The most striking results are with *glc7-L74P*. The *ipl1-2 glc7-L74P* strain grows nearly at the same rate as the wild type (Figure 2D, compare fourth row with first row). These results suggest that the formamide growth defect caused by the *glc7-L74P* allele is connected with Ipl1 activity.

#### SDS22:

**Figure 3:**
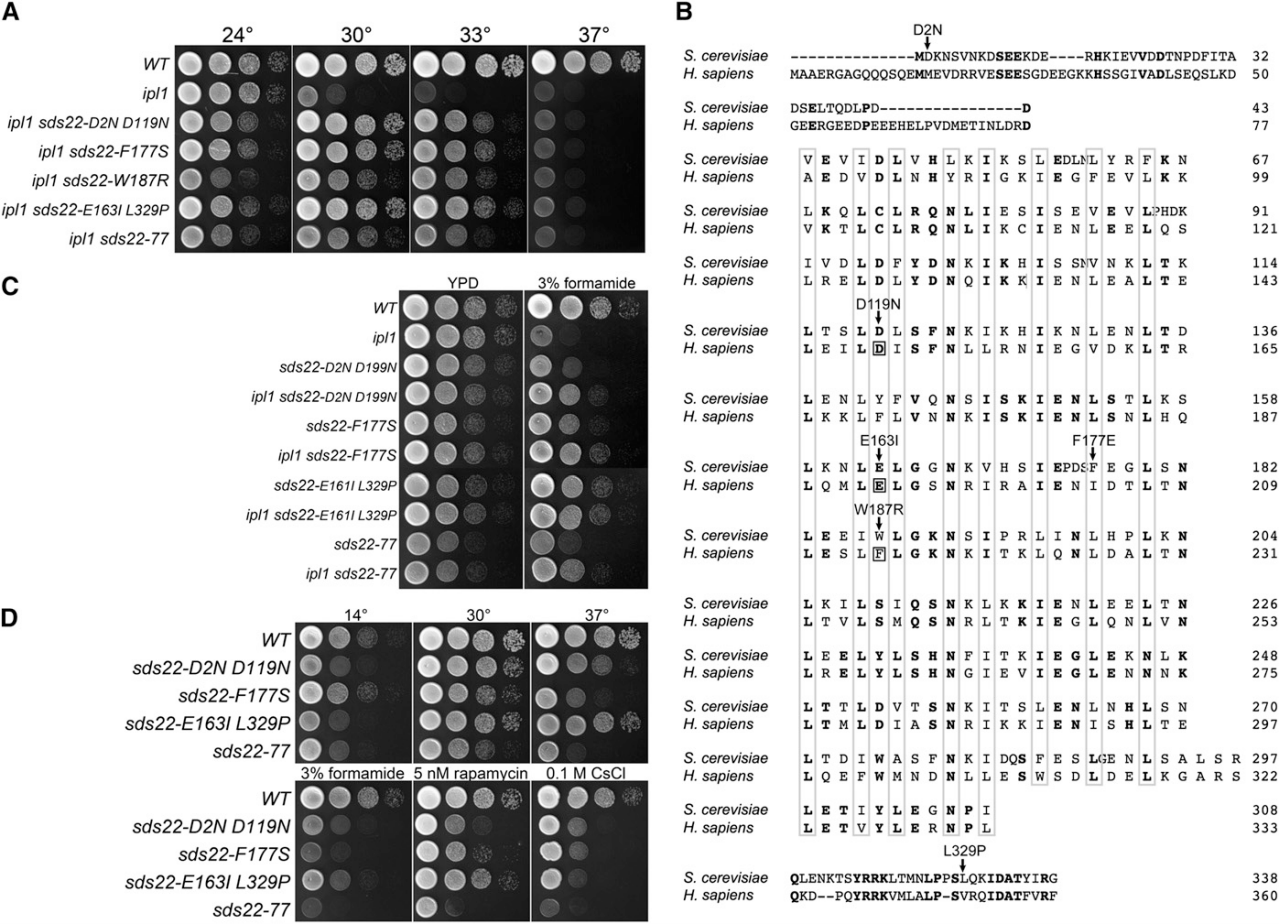
Extragenic *ipl1-2* suppressors in *SDS22*. (A) Cultures of *WT* (KT1113), *ipl1-2* (KT1829), *ipl1-2 sds22-D2N D119N* (KT2934), *ipl1-2 sds22-F177S* (KT3353), *ipl1-2 sds22-W187R* (KT3381), *ipl1-2 sds22-E163I L329P* (KT3052), and *ipl1-2 sds22-77* (KT2936) strains were serially diluted onto YPD medium and imaged after 40 hr at the designated temperatures. (B) ClustalW alignment of human and *S. cerevisiae* Sds22 proteins showing locations of *ipl1* suppressor mutations. Boxed residues correspond to sites of mutations in human Sds22 that prevent proper binding of PP1 (Ceulemans *et al.* 2002). (C) Cultures of WT (KT1113), *ipl1-2* (KT1829), *sds22-D2N D119N* (KT2963), *ipl1-2 sds22-D2N D119N* (KT2934), *sds22-F177S* (KT3351), *ipl1-2 F177S* (KT3353), *sds22-E163I L329P* (KT3292), *ipl1-2 sds22-E163I L329P* (KT3052), *sds22-77* (KT2964), and *ipl1-2 sds22-77* (KT2936) strains were serially diluted onto YPD medium and YPD medium containing 3% formamide and imaged after 24 hr and 72hr at 24°, respectively. (D) Cultures of WT (KT1113), *sds22-D2N D119N* (KT2963), *sds22-F177S* (KT3351), *sds22-E163I L329P* (KT3292), and *sds22-77* (KT2964) strains were serially diluted onto the designated medium at the designated temperatures. Media, with the exception of SC, are either YPD or YPD supplemented as indicated. The YPD 14° plates were incubated for 14 d. All other plates were incubated for 40 hr.

Five suppressors either fail to complement an *sds22* mutant or are linked to *SDS22-mCitrine*::*HIS3* (Figure 3A). *SDS22* encodes a conserved protein whose PP1 binding domain consists of a tandem array of leucine-rich repeats rather than an RVxF motif. Sequence analysis of *SDS22* from these mutants revealed that four contain missense mutations and one results in a frameshift mutation near the 3′ end of the gene. Sequence alignment of Sds22 from budding yeast and human shows that most of the missense mutations are in highly conserved residues (Figure 3B). Four of these, D119, E161, F177E, and W187, are within the leucine-rich repeats. Remarkably, three of these correspond to residues in human Sds22 (D148, E192, F214; boxed residues in Figure 2B) that are required for proper PP1 binding (Ceulemans *et al.* 2002). These observations suggest that Sds22 mutant proteins D119N, E163I L329P and W187R are defective for Glc7 binding.

We were unable to recover *sds22-W187R* in a wild-type *IPL1* background. Spores corresponding to the *sds22-W187R* mutants germinated and grew into microscopic colonies but never formed colonies that could be characterized. Thus, a reduction of Ipl1 activity restores viability to the *sds22-W187R* mutant. This indicates that the inviability of *sds22-W187R* is due to a failure to balance the activity of Ipl1. The other *sds22* mutant alleles are viable in an *IPL1* background, but as observed for some *glc7* mutant alleles, the *sds22ipl1-2* mutants grow better on formamide medium than the corresponding *sds22IPL1* mutants (Figure 3C).

As observed for the *glc7* suppressor mutants, the *sds22* mutants have diverse phenotypes. They exhibit wide variation in sensitivity to rapamycin and 0.1 M CsCl. *sds22-D2N D199N* and *sds22-E163I-L329P* and *sds22*-77 (C-terminal frame shift) confer cold sensitivity (Figure 3D), with accumulation of large budded cells at 14°. *sds22-F117S* and *sds22-77* also cause temperature-sensitive growth. However, unlike the *glc7* suppressor mutations, all *sds22* mutant alleles are completely recessive for *ipl1-2* suppression (Figure S1B).

#### YPI1:

**Figure 4:**
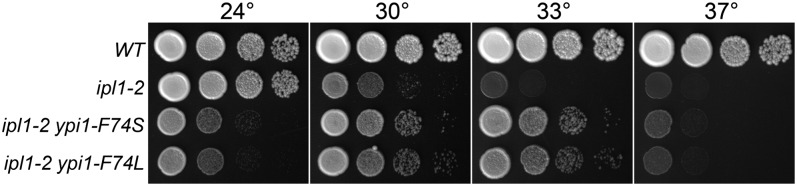
Extragenic *ipl1-2* suppressors in *YPI1*. Cultures of *WT* (KT1113), *ipl1-2* (KT1829), *ipl1-2 ypi1-F74S* (KT3368), and *ipl1-2 ypi1-F74L* (KT3370) strains were serially diluted onto YPD medium and imaged after 40 hr at the designated temperatures.

Two slow-growing *ipl1* suppressors are tightly linked to an *YPI1-13Myc*::*kan* allele. Sequence analysis of the *YPI1* gene in each of these mutants revealed a missense mutation at F74. Revertant 52 has an F74S missense mutation whereas revertant 95 has an F74L mutation. The residue corresponding to F74 in Ypi1/Inh3 orthologs in other eukaryotes is either a phenylalanine or tyrosine. Unlike the *GLC7* mutants, *ypi1-F74S* and *ypi1-F74L* are completely recessive (Figure S1C). *ypi1-F74S ipl1-2* and *ypi1-F74L ipl1-2* mutants grow slowly at room temperature (Figure 4), and these alleles are lethal in a wild-type *IPL1* background. We were unable to recover either *ypi1* mutant in a wild-type *IPL1* background from crosses between the two revertants and a wild-type strain. The spore clones corresponding to the *ypi1IPL1* double mutants germinated and divided several times, but never generated viable colonies. All other genotypes from these crosses exhibited normal viability. The ability of *ipl1-2* to rescue the inviability of *ypi1-F74S* and *ypi1-F74L* suggests that Ypi1, like Sds22, plays an essential function in regulating the Glc7 activity that opposes Ipl1.

#### SHP1:

Revertants 99 and 105 define a complementation group separate from *GLC7*, *SDS22*, and *YPI1*. We assayed for genetic linkage between *rev105* and *SHP1* because *SHP1* was initially identified as a mutant that suppresses the lethality caused by Glc7 overexpression (Zhang *et al.* 1995) and depletion of Shp1 has been shown to suppress the temperature sensitivity of *ipl1-321* (Cheng and Chen 2010). Tetrad analysis between *rev105* (KT3305) and *GFP-CHS4*::*URA3* (KT2684) strains revealed tight linkage between *rev105* and *CHS4/SKT5* (23 PD, 0 TT, 0 NPD; ≤2CM). Because *CHS4* is located 4 kb from *SHP1*, these linkage data strongly support the hypothesis that *rev99* and *rev105* are alleles of *SHP1*. Sequence analysis of the *SHP1* locus from rev99 and rev105 revealed frameshift mutations after codons 122 and 291, respectively. The two mutants are completely recessive for *ipl1-2* suppression (Figure S1D).

Shp1/p47 is a cofactor for Cdc48/p97 (also called VCP in humans), an AAA ATPase that acts as an ATP-dependent chaperone in widely divergent physiological processes. Cdc48 cofactors bind protein substrates that are usually ubiquitinated or have an ubiquitin-like structure and allow the hexameric Cdc48/p97 to structurally remodel the substrate [reviewed in (Jentsch and Rumpf 2007; Meyer *et al.* 2012)]. Shp1 contains an N-terminal 50 aa UBA-like domain that is thought to bind ubiquinated substrate and a C-terminal UBX domain that binds Cdc48. *shp1-99* and *shp1-105* both result in deletion of the UBX domain; thus, their products should not be able to associate with Cdc48. To determine whether *shp1-99* and *shp1-105* behave as null alleles, we constructed a heterozygous *shp1Δ*::*kanMX* mutant in a diploid strain and analyzed the meiotic progeny. Only G418-sensitive clones were recovered, indicating that *SHP1* is an essential gene in our background, as reported for the W303 background (Cheng and Chen 2010). These results indicate that *shp1-99* and *shp1-105* products retain some activity, presumably Cdc48-independent.

**Figure 5:**
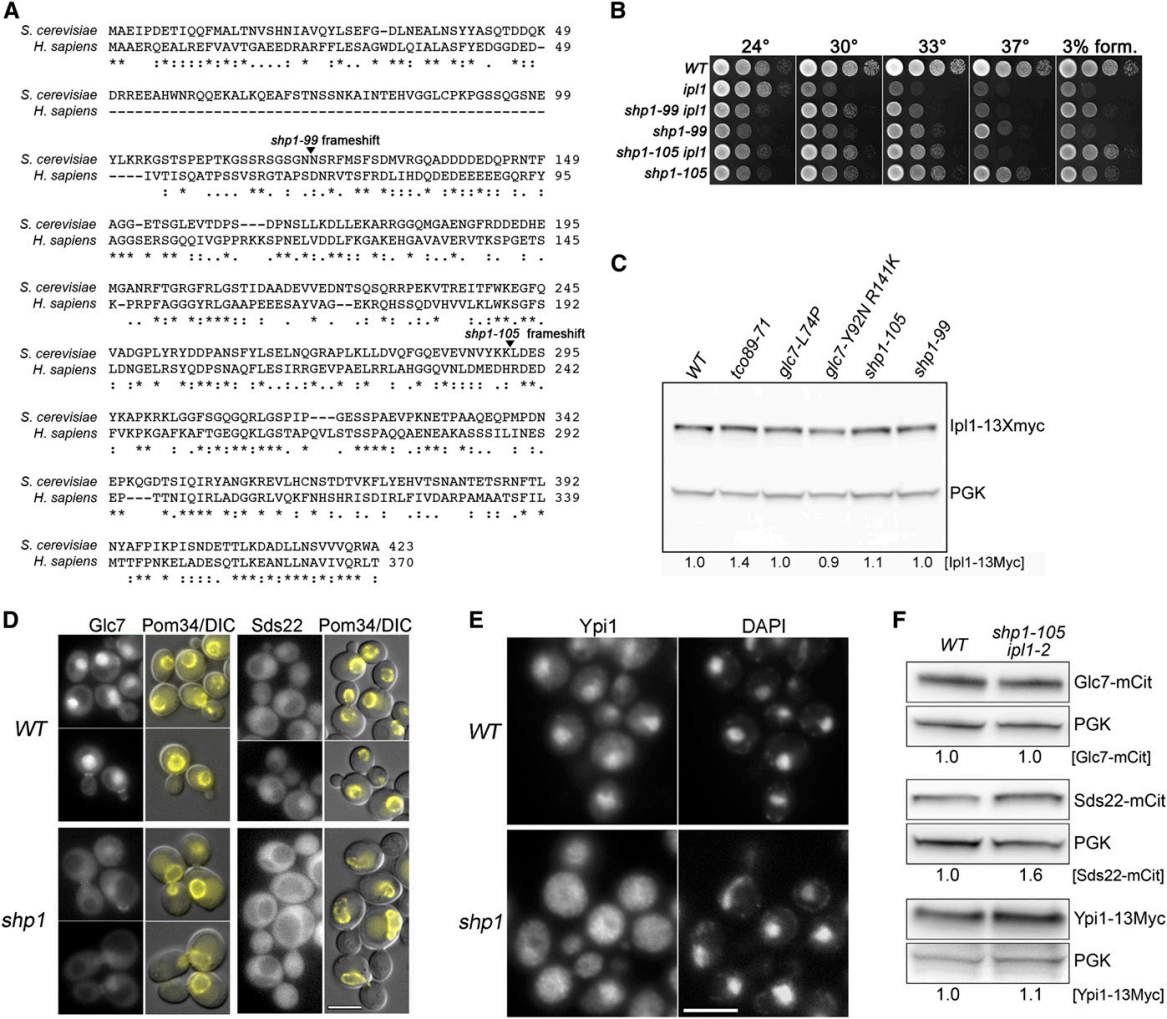
Extragenic *ipl1-2* suppressors in *SHP1*. (A) Sequence alignment of human p47 and *S. cerevisiae* Shp1 proteins with the locations of *ipl1* suppressor mutations indicated. (B) Cultures of *WT* (KT1113), *ipl1-2* (KT1829), *ipl1-2 shp1-99* (KT3413), *shp1-99* (KT3412), *ipl1-2 shp1-105* (KT3419), and *shp1-105* (KT3416) strains were serially diluted onto YPD medium and imaged after 40 hr at the designated temperatures. (C) Immunoblot analysis of extracts from *WT* (KT3383), *tco89-71* (KT3389), *glc7-L74P* (KT3391), *glc7-Y92N R141K* (KT3392), *shp1-105* (KT105), and *shp1-99* (KT3396) strains containing *IPL1-13Myc*. Ipl1-13Myc levels indicated under the lanes were calculated relative to the loading control (Pgk1) signal and normalized to the WT. (D) Fluorescence microscopy of Glc7-mCitrine and Sds22-mCitrine in *WT* (KT3242 and KT2856) and *shp1-105* (KT3424 and KT3428) cells grown to log phase in YPD medium at 24°. The right panels of each set are overlays of Pom34-mCherry and DIC images to demarcate the nuclear periphery. Note that fluorescence images for the Glc7-mCitrine and Sds22-mCitrine are normalized independently. The actual fluorescence levels are much greater in the Glc7-mCitrine strains. Scale bar: 5 μm. (E) Indirect immunofluorescence of Ypi1-13Myc in *WT* (KT2881) and *shp1-105* (KT3427) cells. Scale bar: 5 μm. (F) Immunoblot analysis of extracts from *WT* and *shp1-105 ipl1-2* strains containing Glc7-mCitrine (KT3242 and KT3424), Sds22-mCitrine (KT2856 and KT3428), or Ypi1-13Myc (KT2881 and KT3427). Protein levels were calculated relative to the signal in the loading control (Pgk1) and normalized to the WT.

As is the case for some alleles of *GLC7*, *SDS22*, and *YPI1*, the growth defects of *shp1-99* and *shp1-105* mutant cells are more severe in a wild-type than in an *ipl1-2* background (Figure 5B). This is most striking on YPD medium at 30° or on media containing 3% formamide. Thus, as we have observed for some mutant alleles of *GLC7*, *SDS22*, and *YPI1*, a significant component of the *shp1-99* and *shp1-105* growth defects are associated with *IPL1* function.

Cdc48/p97 has been directly implicated in acting on Aurora B in metazoans. It required for the extraction of Aurora B from mitotic chromatin in *X. laevis* egg extracts and in *C. elegans* embryos (Ramadan *et al.* 2007). In addition, RNAi treatment of two genes encoding Cdc48 (*cdc-48.1* and *cdc-48.2*) alleviates the lethality of a temperature-sensitive Aurora B mutant [*air-2(or207)* (Ramadan *et al.* 2007)]. In contrast, Heallen *et al.* (Heallen *et al.* 2008) identified a Cdc48-related gene (*cdc-48.3*) in an RNAi feeding library screen for suppressors of *air-2(or207)*. However, they found that RNAi of *cdc-48.1* and *cdc48.2*, singly or in combination, did not suppress the lethality of *air-2(or207)*. To determine whether our *SHP1* mutants could be acting to increase Ipl1 levels, we assayed the steady-state levels of Ipl1 by immunoblot analysis in a collection of our *ipl1-2* suppressor mutants. As shown in Figure 5C, Ipl1-13Myc levels are nearly identical between the wild-type strain and *SHP1* mutants, arguing that the *SHP1* suppressor mutants are not acting by increasing Ipl1 protein accumulation.

The levels of nuclear Glc7 are reduced in previously characterized *SDS22*, *YPI1* and *CDC48* mutants (Peggie *et al.* 2002; Pedelini *et al.* 2007; Bharucha *et al.* 2008; Cheng and Chen 2010) and in cells depleted of *YPI* and *SHP1* (Pedelini *et al.* 2007; Bharucha *et al.* 2008; Cheng and Chen 2010). We also reported previously that nuclear Sds22 is decreased in cells depleted of Ypi1 (Bharucha *et al.* 2008). To determine whether loss of Shp1 has similar effects as loss of Ypi1, we imaged Glc7-mCitrine, Sds22-mCitrine, and Ypi1-13Myc in *WT* and *shp1-105* mutants and found that nuclear levels of all three proteins are reduced in the *shp1-105* mutant cells (Figure 5, D and E). Cytoplasmic levels of Sds22 are actually greater than nuclear levels in *shp1* mutant cells (Figure 5D, lower right panels). These results cannot be explained by a decrease in total cellular levels of any of the three proteins. Immunoblot analysis of whole cell extracts (Figure 5F) revealed no obvious change in the total cellular levels of Glc7-mCitrine or Ypi1-13Myc in the *shp1-105* mutant, and Sds22-mCitrine levels are reproducibly higher in *shp1* mutant cells. Immunoblot analysis of two different *shp1-105* strains revealed elevated levels of Sds22-mCitrine (1.6 ± 0.20- and 2.0 ± 0.25-fold, *P* < 0.003 according to the paired Student’s *t*-test).

*SHP1*, *SDS22*, and *YPI1* mutants have remarkably similar effects on Glc7 localization. In all three cases, Glc7 is more uniformly distributed between the nucleus and cytoplasm. Nuclear Sds22 is also reduced in Ypi1 mutants (Bharucha *et al.* 2008), and we show here that *shp1* mutations also affect Sds22 and Ypi1 localization. These effects on Glc7-Ypi1-Sds22 localization are likely through Cdc48 because *cdc48-3* results in the delocalization of Glc7 (Cheng and Chen 2010), and our *shp1* mutant products lack the Cdc48 binding domain. Cdc48 cofactors usually bind substrates that are ubiquitinated or have an ubiquitin-like structure [reviewed in (Jentsch and Rumpf 2007; Meyer *et al.* 2012)]. Glc7 is one possible target in our pathway because it is ubiquitinated *in vivo* (Peng *et al.* 2003; Starita *et al.* 2012). However, the immediate target of Cdc48-Shp1 need not be an ubiquitinated protein, since the ubiquitin-fold protein Atg8 is the substrate for Cdc48-Shp1 in autophagosome biogenesis (Krick *et al.* 2010). Together, these results suggest that the three proteins facilitate nuclear transport or retention of Glc7, a possibility supported by the observation that a screen for dosage suppressors of *ipl1-321* uncovered cytoplasmic Glc7 binding proteins that reduce the nuclear accumulation of Glc7 when overexpressed (Pinsky *et al.* 2006).

### Suppressors in *TCO89*, a component of TORC1

The ability of *rev71* to suppress the temperature sensitivity of *ipl1-2* at 33° does not segregate as a single Mendelian allele. Backcrosses between revertant 71 and an *ipl1-2* strain revealed that, on average, only one of four spore clones grew at 33°, suggesting that two independent mutations are required for growth suppression at 33°. However, two spore clones in each tetrad were cold-sensitive and sensitive to 0.2 mM caffeine in the growth medium. These traits were invariably associated with growth of *ipl1-2* mutants at 30°. We identified this cold and caffeine-sensitive suppressor as a null mutant in *TCO89*, which encodes a component of TORC1. A preliminary characterization of this mutant has been published (Tatchell *et al.* 2011).

We previously proposed that TORC1 acts as a positive regulator of the Glc7 activity that opposes Ipl1 (Tatchell *et al.* 2011). This was based on the observation that mutants with reduced TORC1 levels have lower levels of nuclear Glc7, which has correlated previously with suppression of *ipl1* temperature sensitivity by *SDS22*, *YPI1*, and *SHP1* mutants (Peggie *et al.* 2002; Pedelini *et al.* 2007; Bharucha *et al.* 2008; Cheng and Chen 2010). We also noted genetic interactions between mutants in the TORC1 pathway and *GLC7* mutants that suppress *ipl1-2*. Among three alleles of *GLC7* that were tested previously for suppression of *ipl1-2* (Hsu *et al.* 2000), the two that acted as strong suppressors of *ipl1-2* (*glc7-127* and *glc7-129*) grow slowly in the presence of TORC1 inhibitors rapamycin or caffeine, whereas the *glc7-109* mutation, which was previously shown to poorly suppress the temperature sensitivity of *ipl1-2*, does not confer strong sensitivity to rapamycin and caffeine (Tatchell *et al.* 2011). Our collection of 10 new *GLC7* mutants that suppress the temperature sensitivity of *ipl1-2* provided us with a more complete panel to test the relationship between Glc7 and TORC1. As shown in Figure 2C, there was significant variability in the sensitivity of the different *GLC7* alleles to rapamycin. The sensitivity to rapamycin and caffeine conferred by *GLC7* alleles correlates with negative genetic interactions with TORC1 mutants. For example, the rapamycin-resistant *glc7-L74P* mutant does not exhibit a strong genetic interaction with *tco89Δ*::*kanMX*, whereas the more rapamycin-sensitive *glc7-L37F* mutant is nearly lethal in combination with the *tco89Δ*::*kanMX* mutation (Figure S2). However, there is no strong correlation between the ability of a *GLC7* allele to suppress *ipl1-2* and the level of sensitivity to rapamycin conferred by that allele. For example, *glc7-L74P* is a strong suppressor of *ipl1-2* (Figure 2B) but confers resistance to rapamycin (Figure 2C) and caffeine (data not shown). In contrast, many other *GLC7* mutant alleles are less effective *ipl1-2* suppressors but are more sensitive to rapamycin than *glc7-L74P*. The complexity of these genetic interactions suggests that TORC1 does not act simply as a positive regulator of total cellular Glc7 activity. Further characterization of these different *GLC7* alleles may provide clues to the mechanism of TORC1 influence on nuclear Glc7 activity.

### *ipl1-2* suppressors in components of the kinetochore

#### DUO1, a component of the Dam1/DASH kinetochore complex:

**Figure 6:**
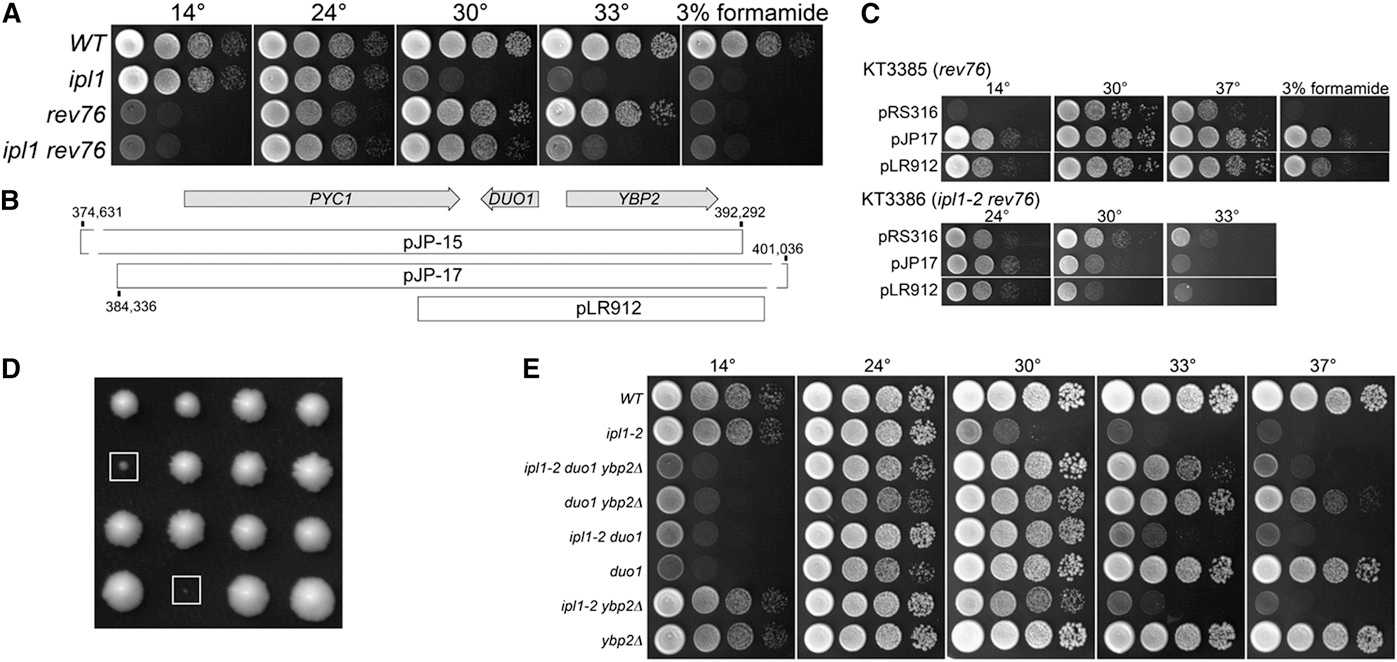
Extragenic *ipl1-2* suppressors in *DUO1*. (A) Cultures of *WT* (KT1113), *ipl-2* (KT1829), *rev76* (KT3385), and *ipl1-2 rev76* (KT3386) strains were serially diluted onto YPD medium and imaged after 40 hr at the indicated temperatures. (B) Genetic map of the *PYC1-DUO1-YBP2* region of chromosome VII showing the locations of genomic DNA fragments that complement the *rev76* suppressor phenotype. (C) Cultures of *duo1-S115F* (KT3385) and *ipl1-2 duo1-S115F* (KT3386) mutant strains transformed with the designated plasmids were serially diluted onto the following media: Top panels: YPD at the designated temperatures and YPD + 3% formamide at 30°. Bottom panels: Synthetic medium lacking uracil at the designated temperatures. (D) Images of tetrads from a cross between *duo1-S115F* and *mad1*::*HIS3* strains (KT3386 X KT1688). The boxes identify the *duo1-S115F mad1*::*HIS3* double mutants. These were the largest double mutant colonies observed; the majority of double mutants failed to grow into macroscopic colonies. Each column represents the four spore clones of a tetrad. (E) Cultures of *WT* (KT1113), *ipl-2* (KT1829), *ipl1-2 duo1-S115F ybp2Δ*::*kanMX6* (KT3409), *duo1-S115F ybp2Δ*::*kanMX6* (KT3410), *ipl1-2 duo1-S115F* (KT3386), *duo1-S115F* (KT3385), *ipl1-2 ybp2Δ*::*kanMX6* (KT3401), and *ybp2Δ*::*kanMX6* (KT3403) strains were serially diluted onto YPD medium and imaged after 40 hr at the indicated temperatures with the exception of the plate at 14°, which was imaged after 7 d.

Revertants 76 and 81 are cold-sensitive, arresting as large budded cells at 14°, and are sensitive to 3% formamide at all temperatures (Figure 6A). The suppressor mutations in these two mutants are unlinked to *GLC7*, *YPI1*, *SHP1*, and *SDS22*. The cold and formamide sensitivities of revertants 76 and 81 failed to complement. To identify this locus, we transformed each mutant with a low-copy yeast genomic library and selected for growth in medium containing 3% formamide. The identities of the genomic inserts in plasmids conferring formamide resistance were determined by sequence analysis.

The genomic inserts in two clones that complement both cold and formamide sensitivities of revertants 76 and 81 contain overlapping regions of chromosome VII. The region of overlap contains *PYC1*, encoding pyruvate carboxylase, *DUO1*, encoding a component of the DAM/DASH kinetochore complex, and *YBP2*, a component of the kinetochore that is required for normal mitotic progression [Figure 6B (Ohkuni *et al.* 2008)]. To confirm that the *rev76* and *rev81* loci are genetically linked to this region of chromosome VII, we determined the map distance between *rev76* and *rev81* and two neighboring genes (*pyc1Δ*::*kanMX* and *pkp2Δ*::*kanMX)*. In neither case was recombination observed between formamide sensitivity and the G418 resistance associated with *pyc1Δ*::*kanMX and pkp2Δ*::*kanMX* (≤ 2 map units for both crosses). Plasmid pLR912, containing *DUO1* and *YBP2* in yeast shuttle vector pRS316, fully complements the cold and formamide sensitivities as well as the *ipl1-2* suppression phenotype of *rev76*. Sequence analysis of *DUO1* from rev76 and rev81 revealed two base substitutions, C344T and C607T, that result in missense mutations S115F and P203S. C607T also was found in our wild-type strain. S115 is highly conserved in other sensu stricto yeasts and in Duo1 from *S. pombe*, but P203 is not conserved. In fact, other Duo1 proteins from sensu stricto yeasts contain an S or T at residue 203. The sequences of *YBP2* obtained from rev76, rev81, and our wild-type strain are identical, but each differs from the S288C wild-type strain at six nucleotides, resulting in three missense mutations (H136Q, K140N, and I286T) and three silent mutations. These polymorphisms were previously reported to occur in a different genetic background (Ohkuni *et al.* 2008). H136, K140, and I286 are not conserved among sensu stricto yeasts, and Ohkuni *et al.* (Ohkuni *et al.* 2008) state that no functional significance of any was observed. Thus, the *ipl1* suppressor mutation in *rev76* and *rev81* is most likely C344T in *DUO1*, causing the S115F change. Because rev76 and rev81 were isolated from the same round of mutagenesis and have identical phenotypes and identical DNA sequences at *DUO1*, the two revertants are likely from the same mutagenic event. Hereafter, we refer to the *rev76* and *rev81 ipl1* suppressors as *duo1-S115F*. To confirm that *duo1-S115F* is responsible for *ipl1-2* suppression, we mapped the *ipl1-2* suppressor in rev76 to the *DUO1* region of chromosome VII as described in the *Materials and Methods*. We observed no recombination between the *ipl1-2* suppression in rev76 and *ybp2Δ*::*kan* in 33 tetrads (<1.5 map units). We also replaced the wt *DUO1* gene with the *duo1-S115F* allele from rev76 (*Materials and Methods*). All spore clones containing *ipl1-2* and *duo1-S115F* grow at 30°, whereas *ipl1-2 DUO1* clones cannot (Figure S3A).

*duo1-S115F* mutant cells arrest as large budded cells at 14°. A likely explanation for this arrest is that the *duo1* mutation activates the SAC, delaying cells at the metaphase-anaphase transition. To test this possibility, we crossed a *duo1-S115F* mutant to the SAC mutant *mad1*::*HIS3* and analyzed the progeny. Due to genetic linkage between *MAD1* and *DUO1*, we identified only 27 tetrads predicted to contain the *mad1*::*HIS3duo1-S115F* double mutant from a total of 120 tetrads (map distance 11.2 map units). Only four of these predicted double mutants grew into small but visible colonies (Figure 6D). Sixteen formed microcolonies containing between 2 and ∼100 cells and seven failed to germinate or divide. These results are consistent with *duo1-S115F* activating the SAC.

Duo1 is 1 of 10 protein components of the microtubule binding Dam1 or DASH complex. The complex forms rings around microtubules *in vitro* (Miranda *et al.* 2005; Westermann *et al.* 2005) and can track the plus ends of microtubules (Asbury *et al.* 2006; Westermann *et al.* 2006; Gestaut *et al.* 2008), although the ring structure is not necessarily required for end tracking (Gestaut *et al.* 2008). Dam1/DASH also increases the processivity of the NDC80 complex (Lampert *et al.* 2010; Tien *et al.* 2010). The Duo1 and Dam1 subunits are thought to form the primary microtubule-binding domain of the complex (Miranda *et al.* 2007). Dam1 is phosphorylated by Ipl1/Aurora B, which decreases the affinity of the Dam1 complex for microtubules (Gestaut *et al.* 2008), but Duo1 is not known to be a substrate for Ipl1. Two previously characterized temperature-sensitive *DUO1* mutants (*duo1-1* and *duo1-2*) arrest with short spindles at the nonpermissive temperature due to activation of the SAC (Hofmann *et al.* 1998) and one of these (*duo1-2*) contains two missense mutations (A117T M124I) close to the mutation in *duo1-S115F*. Rather than suppress the temperature sensitivity of *ipl1* mutations, however, the *duo1-2* mutation was observed to be lethal in combination with *ipl1-1* and showed no genetic interaction with *ipl1-2* (Kang *et al.* 2001).

How is Duo1-S115F altering the function of the Dam1/DASH complex? Although Duo1 phosphorylation has not been reported, it is possible that S115 is phosphorylated, or that the mutation increases the phosphorylation state of other Dam1/DASH subunits. Alternatively, the altered Duo1 product may interact differently with the complex to affect the regulation of microtubule-binding.

#### ybp2Δ suppresses the temperature sensitivity of ipl1-2:

*YBP2* lies adjacent to *DUO1* and is transcribed from the opposite strand. *YBP2* was originally identified because of its sequence similarity to *YBP1*, which is involved in the oxidative stress response (Gulshan *et al.* 2004). More recently, *YBP2* was identified based on its negative genetic interaction with *MAD2* (Ohkuni *et al.* 2008). Strains deleted for *YBP2* show increased chromosome loss, and, in immune complexes, Ybp2 was reported to associate with Ndc80, MIND and COMA kinetochore components, as well as the centromere-specific histone Cse4 (Ohkuni *et al.* 2008). Paradoxically, the association of several kinetochore components with one another and with the centromere appears to be enhanced in a *ybp2* null mutant (Ohkuni *et al.* 2008). In the course of our analysis of *DUO1* and *YBP2*, we found that a *ypb2Δ*::*kanMX* mutation suppresses the temperature sensitivity of *ipl1-2* at 30° (Figure 6E, row 7). Furthermore, the *ybp2Δ*::*kanMX* mutation enhances the ability of a *duo1-S115F* mutant to suppress *ipl1-2* (Figure 6E, row 3) and *ybp2Δ*::*kanMX duo1-S115F* double mutants grow slowly at 37° (Figure 6E, row 4). We also observed genetic interactions between *ybp2Δ*::*kanMX* and *mad1*::*HIS3*. Analysis of a cross between *ybp2Δ*::*kanMX* and *mad1*::*HIS3* strains (KT3400 × KT1687) revealed that all *ybp2Δ*::*kanMX mad1*::*HIS3* double mutant spore clones were small or microcolonies (Figure S4). Spore clones of all other genotypes grew normally. Thus, as previously reported for another genetic background, loss of Ybp2 in our genetic background is not lethal, but results in SAC activation. Although Ybp2 apparently has a positive role in kinetochore function, the enhancement of interactions among NDC80, MIND, and COMA complex components in the absence of Ybp2 is puzzling. The suppression of *ipl1-2* by loss of *YBP2* is consistent with a positive role in kinetochore function, and suggests that whatever enhancement of complex interactions occurs in the deletion mutant, the net effect may be destabilization of kinetochore-microtubule interaction. This would be consistent with the chromosome loss phenotype of the deletion mutant.

#### Revertant 8 is a missense mutation in NDC80:

Revertant 8 *(*rev8*)* is hypersensitive to formamide and arrests with large buds at low temperature but is complemented by *duo1-S155F*. We isolated four clones from two genomic libraries that complement the formamide and cold sensitivity of rev8. The four clones contain overlapping regions of chromosome IX. The only full-length gene in common among the four clones is the evolutionarily conserved *NDC80/TID3* gene. To confirm that the mutation in rev8 responsible for *ipl1-2* suppression is genetically linked to the *NDC80* locus, we determined the map distance between the *ipl1-2* suppressor in *rev8* and *NDC80* tagged with *3XHA*::*KanMX* and *NDC80-3XFlag*::*KanMX*. No recombination was observed between *rev8* and the tagged *NDC80* alleles in 56 tetrads (≤0.9 map units), indicating that *rev8* is likely an *NDC80* mutant. Ndc80/HEC1 is one of four subunits in the Ndc80 complex, the major microtubule binding and end tracking component of the kinetochore. The N-terminal portions of Ndc80/HEC1 and Nuf1 subunits bind microtubules, while their C-terminal coiled-coil domains associate with coiled-coil domains of the Spc24 and Spc25 subunits, which tether the complex to the inner kinetochore [reviewed by (DeLuca and Musacchio 2012)].

**Figure 7:**
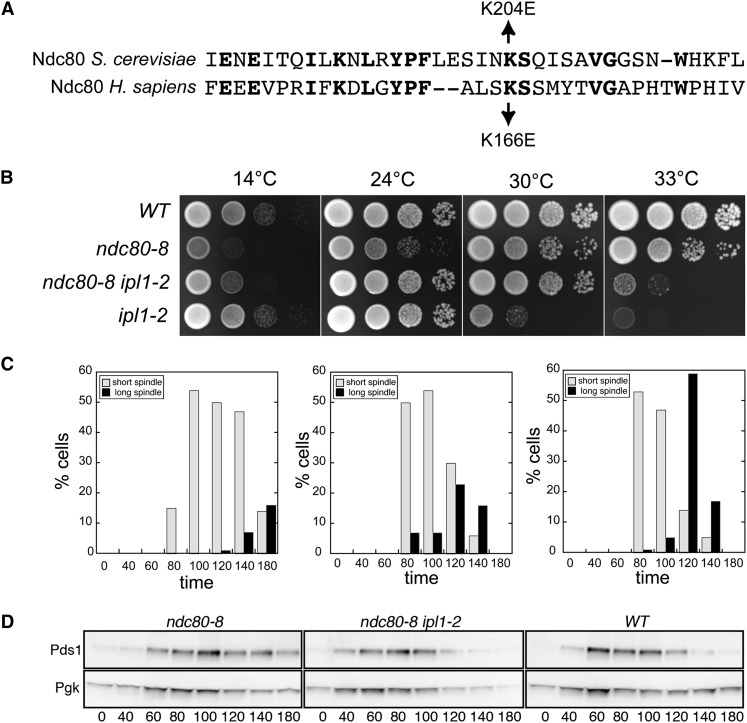
Extragenic *ipl1-2* suppressors in *NDC80*. (A) Sequence alignment of yeast and human Ndc80 proteins. (B) Cultures of *WT* (KT1113), *ndc80-K204E (ndc80-8) (KT3255)*, *ndc80-K204E ipl1-2* (KT3257), and *ipl1-2 (KT1963)* strains were serially diluted, plated onto indicated media, and imaged after 44 hr. The first four panels show YPD medium incubated at the designated temperatures. (C) and (D) Cultures of *ndc80-K204E(KT3317)*, *ndc80-K204E ipl1-2 (KT3320)*, and WT (KT3319) cells were arrested with alpha factor, released into YPD medium at 24°, and monitored for spindle length and entrance into anaphase using Spc42-3XGFP and Pds1-13Myc fusions, respectively, at the designated time points. (C) Quantitation of the percentage of cells with short (≤2.0 μm) or long (>2.0 μm) spindles. At least 64 mitotic spindles were measured at each time point for each strain. (D) Immunoblot analysis of Pds1-13XMyc in whole cell extracts. Pgk serves as the loading control.

Sequence analysis of *NDC80* from a rev8 strain revealed a single nucleotide substitution in *NDC80*, resulting in missense mutation K204E. K204 corresponds to K166 in human Ndc80/Hec1 (Figure 7A) and K89 in human EB1, which lies in the microtubule-binding calponin-homology (CH) domain. The K166E mutation in human Ndc80 and K89E in EB1 strongly impairs or eliminates *in vitro* microtubule binding, respectively (Hayashi and Ikura 2003; Ciferri *et al.* 2008), indicating that this conserved lysine residue in the N-terminal domain of Ndc80 is required for efficient microtubule binding. *In vivo*, Ndc80/Hec1 mutant cells containing K166D and K166E substitutions fail to accumulate in metaphase and have weak kinetochore-microtubule attachments (Sundin *et al.* 2011; Tooley *et al.* 2011). To confirm that the *ipl1-2* suppressor in rev8 is ndc80-K204E, we replaced the wt *NDC80* gene with a cloned version of *NDC80* from rev8 see Materials and Methods). All *ipl1-2* spore clones containing *ndc80-K204E* grew at 30° whereas those containing wt *NDC80* did not (Figure S3B).

*ndc80-K204E* mutant cells arrest with large buds and short mitotic spindles at 14° (data not shown). Interestingly, the slow growth phenotype of *ndc80-K204E* at 14° and 24° is suppressed by *ipl1-2* (Figure 7B). In a reciprocal manner, the temperature sensitivity of *ipl1-2* is suppressed by *ndc80-K204E* at 30° and partially at 33° (Figure 7B). To characterize the slow growth phenotype of *ndc80-K204E* cells in more detail, we arrested *ndc80-K204E*, *ipl1-2 ndc80-K204E*, and *WT* strains in G1 with alpha mating pheromone, and monitored spindle formation and anaphase progression after cell cycle release in the absence of mating pheromone. Mating pheromone was added to the cultures after 60 min to prevent cells from re-entering the cell cycle. The strains contained *SPC42-3XGFP* and *PDS1-13XMyc* to facilitate monitoring spindle pole distances and entrance into anaphase, respectively. Wild-type cells duplicated their spindle pole bodies and formed spindles after 60 min. A majority of the cells were in anaphase by 120 min (Figure 7C, right panel), as shown by both spindle length and decreased Securin/Pds1 levels (Figure 7D, right panel). At 180 min, all wild-type cells had exited mitosis, as shown by unduplicated spindle pole bodies and the absence of Pds1. Although *ndc80-K204E* mutant cells started forming spindles after 60 min, less than 20% of the cells had entered anaphase at 180 min and substantial Pds1 remained in the cells by 180 min, consistent with an extended G2/M cell cycle delay (Figure 7, C and D, left panels). The delay is largely eliminated in *ndc80-K204E ipl1-2* mutant cells (Figure 7, C and D, middle panels); all cells returned to G1 by the 180-minute time point.

Given the observation that the human Ndc80-K166E variant protein binds weakly to microtubules *in vitro*, it is likely that the cell cycle delay in *ndc80-K204E* cells is due to SAC activation. Consistent with this possibility, although we recovered slow growing *ndc80-K204E mad1*::*HIS3ipl1-2* triple mutants, we failed to recover viable *ndc80-K204E mad1*::*HIS3* progeny from a cross between *ndc80-K204E ipl1-2* and *mad1*::*HIS3* strains. These results strengthen the evidence that loss of Ipl1 activity can compensate for the loss of kinetochore-microtubule binding.

The identification of the *ndc80-K201E* mutant as an *ipl1-2* suppressor represents a remarkable convergence of genetics, cell biology, biochemistry, and structural analysis. Previous studies have shown that basic residues in the unstructured N-terminus and in the CH domain of Ndc80 bind microtubules via electrostatic interactions between the Ndc80 complex and microtubules. Phosphorylation of the N-terminus of Ndc80 by AuroraB/Ipl1 is thought to reduce the negative charge of the microtubule binding domain and thus increase the microtubule/kinetochore dynamics necessary for the dissolution of improper kinetochore-microtubule interactions. Consistent with this model, the Ndc80-7A mutant, in which Aurora B phosphorylation sites in the N terminus of Ndc80 are mutated to alanine, is inviable under conditions of reduced Ipl1 activity [*ipl1-321* background (Akiyoshi *et al.* 2009a)]. Our results provide direct biological support for the hypothesis that the phosphorylation of Ndc80 by Ipl1/Aurora B reduces the affinity of the Ndc80 complex for kinetochore microtubules, thereby increasing the kinetochore/microtubule dynamics required to effect bipolar chromosome attachment on the spindle.

In addition to three intragenic suppressors of *ipl1-2*, our classical genetic screen identified new alleles in previously identified components of the Glc7/PP1 pathway (*GLC7*, *YPI1*, *SDS22*, and *SHP1*), a component of the TORC1 complex (*TCO89*) and two mutants in components of the outer kinetochore (*DUO1* and *NDC80*). Given the considerable attention *IPL1* has received, and the plethora of genome-based screening strategies, we think that it is worth comparing our genetic screen with others, both directed and undirected. First, we did not recover mutants in *GLC8*, for which null alleles are known to suppress temperature sensitive *IPL1* mutants (Tung *et al.* 1995). It is likely that the 33° temperature we set for the screen was too high to recover *GLC8* mutants since *glc8* null mutants in our background only suppress the *ipl1-2* mutant allele weakly at 33° (Tatchell *et al.* 2011). It is possible that 33° was also too stringent to recover mutants in components of the Set1 protein methylation complex that methylates Dam1, which were previously shown to suppress *ipl1-2* (Zhang *et al.* 2005; Latham *et al.* 2011). We note that *tco89-71* also suppresses weakly at 33° but in this case, the original suppressor strain contained a second mutation that enhanced the suppression at 33°. In the future, it might be possible to sensitize the screen by using a genetic background that contains a weak suppressor.

With the exception of *TCO89*, all of the suppressor mutations identified in our screen are in evolutionarily conserved, essential genes, underscoring the continued value of traditional genetic screens. A high throughput screen of the deletion collection would have missed key components of the pathway. The screen is clearly not saturated, since only one allele each of *DUO1* and *NDC80* was identified, and we did not isolate any mutations in *YBP2*, which we found serendipitously to be an *ipl1* suppressor locus. Given the potential importance of these novel alleles, it could be informative to repeat the screen with a starting strain in which *GLC7* and *SDS22* were duplicated, in order to reduce or eliminate the frequency of mutants in the Glc7 pathway.

## Supplementary Material

Supporting Information
