# Supplementary material for: Suppressors of ipl1-2 in Components of a Glc7 Phosphatase Complex, Cdc48 AAA ATPase, TORC1, and the Kinetochore
Source: G3 (Bethesda). 2012 Dec 1;2(12):1687–701. doi: 10.1534/g3.112.003814 (PMC3516489; doi:10.1534/g3.112.003814)
Supplement: Supporting Information [file supp_2_12_1687__index.html]

Supporting Information 

# Suppressors of *ipl1-2* in Components of a Glc7 Phosphatase Complex, Cdc48 AAA ATPase, TORC1, and the Kinetochore

## Supporting Information for Robinson *et al.*, 2012

**Files in this Data Supplement:**

- Supporting Information - Figures S1-S4 and Tables S1 and S2 (PDF, 5.4 MB)
- Figure S1 - Dominance of *ipl1* suppressors (PDF, 1.9 MB)
- Figure S2 - Genetic interactions between *GLC7* mutant alleles and *TCO89* (PDF, 485 KB)
- Figure S3 - Confirmation of *duo1-S155F* and *ndc80-K204E* alleles (PDF, 2.1 MB)
- Figure S4 - Genetic interactions between *ybp2Δ* and *mad1Δ* mutant alleles (PDF, 1.1 MB)
- Table S1 - Strain list (PDF, 118 KB)
- Table S2 - Primers Used (PDF, 77 KB)
