# Supplementary material for: Suppressors of ipl1-2 in Components of a Glc7 Phosphatase Complex, Cdc48 AAA ATPase, TORC1, and the Kinetochore
Source: G3 (Bethesda). 2012 Dec 1;2(12):1687–701. doi: 10.1534/g3.112.003814 (PMC3516489; doi:10.1534/g3.112.003814)
Supplement: Supporting Information [file supp_2.12.1687_FigureS1.pdf]

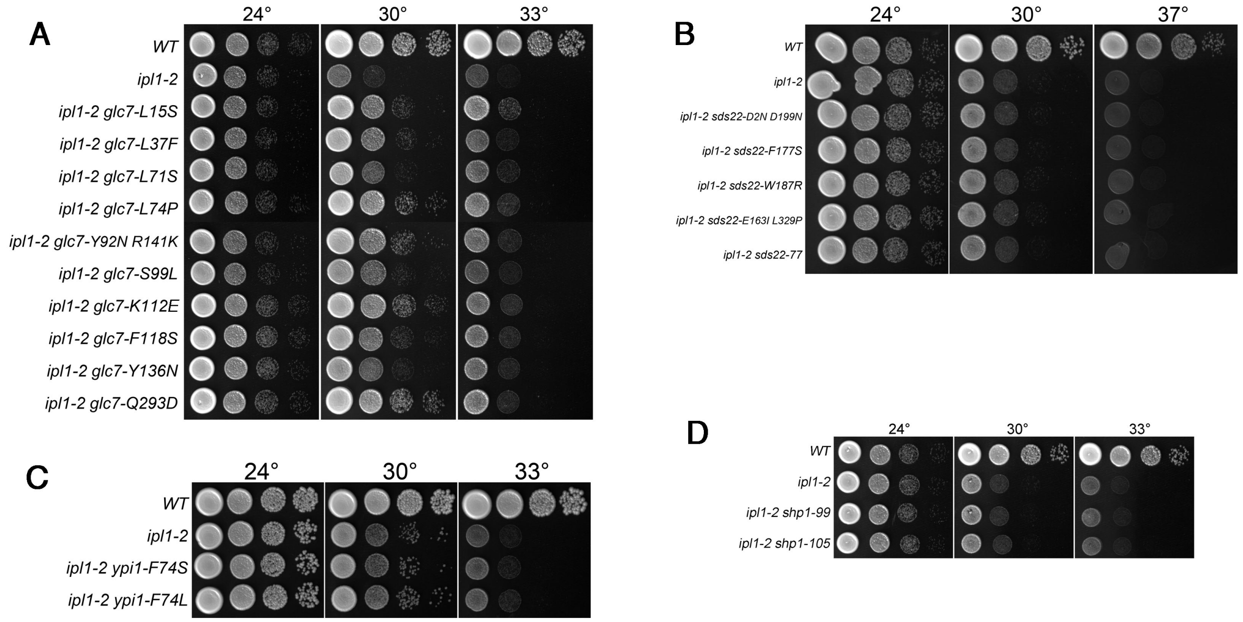

Figure S1 Dominance of *ipl1* suppressors. Cultures of diploid strains homozygous for *ipl1-2* and heterozygous for the designated *ipl1-2* suppressor mutation were serially diluted onto YPD medium and imaged after 40 hr at the designated temperatures. The WT strain is heterozygous for *ipl1-2* (KT1112 X KT1829). Suppressor mutations in *GLC7*, *SDS22*, *YPI1* and *SHP1* are presented in panels (A), (B), (C), and (D), respectively. Note that only the *GLC7* mutant alleles display semi-dominance.
