# Supplementary material for: Suppressors of ipl1-2 in Components of a Glc7 Phosphatase Complex, Cdc48 AAA ATPase, TORC1, and the Kinetochore
Source: G3 (Bethesda). 2012 Dec 1;2(12):1687–701. doi: 10.1534/g3.112.003814 (PMC3516489; doi:10.1534/g3.112.003814)
Supplement: Supporting Information [file supp_2.12.1687_FigureS2.pdf]

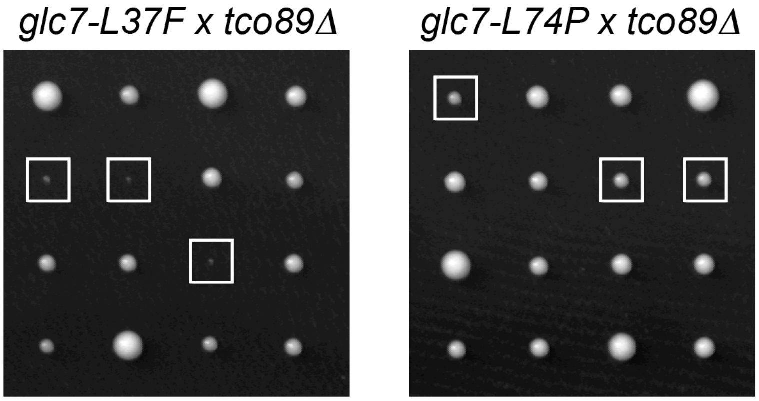

Figure S2 Genetic interactions between *GLC7* mutant alleles and *TCO89*. Images of four tetrads from a cross between *glc7-L37F* (left panel) and *glc7-L74P* (right panel) and a *tco89Δ::kanMX* strain. The boxes identify the *glc7 tco89Δ* double mutants. Each column represents the four spore clones of a tetrad. Note that the rapamycin-sensitive *GLC7* mutant (*glc7-L37F*) exhibits the strongest genetic interaction with *tco89Δ.*
