# Supplementary material for: Suppressors of ipl1-2 in Components of a Glc7 Phosphatase Complex, Cdc48 AAA ATPase, TORC1, and the Kinetochore
Source: G3 (Bethesda). 2012 Dec 1;2(12):1687–701. doi: 10.1534/g3.112.003814 (PMC3516489; doi:10.1534/g3.112.003814)
Supplement: Supporting Information [file supp_2.12.1687_FigureS3.pdf]

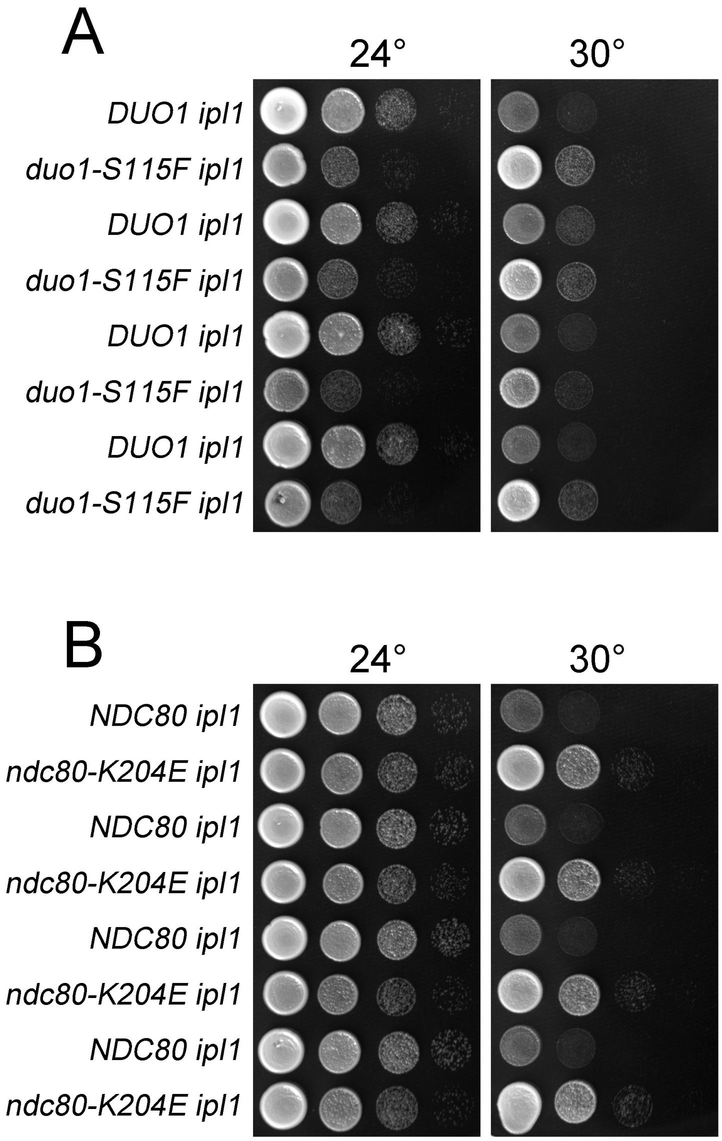

Figure S3 Confirmation of *duo1-S155F* and *ndc80-K204E* alleles. Cultures of ascospore clones were serially diluted onto YPD medium and imaged after 28-30 hr at the designated temperatures. (A) Haploid spore clones from a diploid strain KT3349 (*ipl1-2/IPL1 duo1Δ::kan/DUO1* pRS303:duo1-S115F). All the clones are *ipl1-2* and contain either wild-type *DUO1* or pRS303:*duo1-S115F*. As expected the pRS303-*duo1-S115F* clones are also cold sensitive, arresting as large budded cells at 14°. (B) Haploid spore clones from a diploid strain KT3350 (*ipl1-2/IPL1 ndc80Δ::kan/NDC80* pRS316:*ndc80-K204E*). All the clones are *ipl1-2* and contain either wild-type *NDC80* or pRS316:*ndc80-K204E.*
