# Supplementary material for: Suppressors of ipl1-2 in Components of a Glc7 Phosphatase Complex, Cdc48 AAA ATPase, TORC1, and the Kinetochore
Source: G3 (Bethesda). 2012 Dec 1;2(12):1687–701. doi: 10.1534/g3.112.003814 (PMC3516489; doi:10.1534/g3.112.003814)
Supplement: Supporting Information [file supp_2.12.1687_FigureS4.pdf]

ybp2Δ x mad1Δ

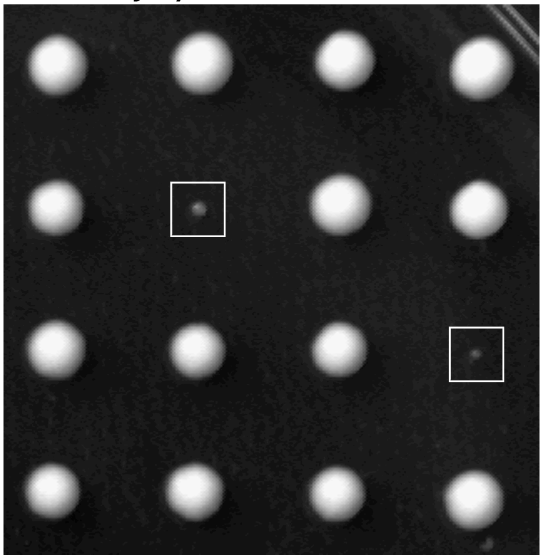

Figure S4 Genetic interactions between *ybp2Δ and mad1Δ* mutant alleles. Images of four tetrads from a cross between *ybp2Δ::kanMX* and a *mad1Δ::HIS3* strain. The boxes identify the *ybp2Δ mad1Δ double mutants.* Each column represents the four spore clones of a tetrad.
