# Supplementary material for: Suppressors of ipl1-2 in Components of a Glc7 Phosphatase Complex, Cdc48 AAA ATPase, TORC1, and the Kinetochore
Source: G3 (Bethesda). 2012 Dec 1;2(12):1687–701. doi: 10.1534/g3.112.003814 (PMC3516489; doi:10.1534/g3.112.003814)
Supplement: Supporting Information [file supp_2.12.1687_TableS1.pdf]

Table S1 Strain list

strain	genotype	Source or reference
KT1112	*MAT***a** *leu2 ura3 his3*	(Stuart et al., 1994)[a]
KT1113	*MAT*α *leu2 ura3 his3*	(Frederick and Tatchell, 1996)[b]
KT1687	*MAT*α *mad1::HIS3*	this study
KT1688	*MAT***a** *mad1::HIS3*	this study
KT1829	*MAT*α *ipl1-2 (H352Y)*	(Hsu et al., 2000)[c]
KT1963	*MAT***a** *ipl1-2*	(Bharucha et al., 2008)[d]
KT2856	*MAT***a** *SDS22-mCitrine::SpHis5 POM34-mCherry::SpHis5*	this study
KT2865	*MAT***a** *ipl1-R150K H352Y*	this study
KT2867	*MAT***a** *ipl1-S167L H352Y*	this study
KT2869	*MAT***a** *ipl1-G347E H352Y*	this study
KT2878	*MAT***a** *ipl1-2 ndc80-K204E ura3::GFP-TUB1::URA3*	this study
KT2881	*MAT***a** *YPI1-13Myc:kanMX6*	this study
KT2934	*MAT*α *ipl1-2 sds22-D2N D119N*	this study
KT2936	*MAT*α *ipl1-2 sds22-77(Cterm frameshift)*	this study
KT2938	*MAT*α *ipl1-2 glc7-L71S*	this study
KT2939	*MAT*α *ipl1-2 glc7-S99L*	this study
KT2940	*MAT*α *ipl1-2 glc7-L37F*	this study
KT2963	*MAT*α *sds22-D2N D119N*	this study
KT2964	*MAT*α *sds22-77*	this study
KT2961	*MAT*α *duo1-S115F (rev76)*	this study
KT2967	*MAT*α *duo1-S115F (rev81)*	this study
KT2969	*MAT*α *glc7-L71S*	this study
KT2970	*MAT*α *glc7-S99L*	this study
KT2973	*MAT*α *glc7-L37F*	this study
KT3052	*MAT*α *ipl1-2 sds22-E163I L329P*	this study

KT3059	*MATα ipl1-2 glc7-F118S*	this study
KT3062	*MATα ipl1-2 glc7-L15S*	this study
KT3064	*MATα ipl1-2 glc7-K112E*	this study
KT3066	*MATα ipl1-2 glc7-Q293D*	this study
KT3242	*MAT***a** *GLC7-mCitrine:SpHis5 POM34-mCherry:SpHis5*	(Tatchell et al., 2011)[e]
KT3255	*MAT***a** *ndc80-K204E*	this study
KT3257	*MAT***a** *ipl1-2 ndc80-K204E*	this study
KT3292	*MATα sds22-E163I L329P*	this study
KT3302	*MATα glc7-F118S*	this study
KT3304	*MATα glc7-L15S*	this study
KT3308	*MATα glc7-K112E*	this study
KT3310	*MATα glc7-Q293D*	this study
KT3317	*MAT***a** *PDS1-13Myc:kanMX6 SPC42-3XGFP:TRP1 bar1-1 ndc80-K204E*	this study
KT3319	*MAT***a** *PDS1-13Myc:kanMX6 SPC42-3XGFP:TRP1 bar1-1*	this study
KT3320	*MAT***a** *PDS1-13Myc:kanMX6 SPC42-3XGFP:TRP1 bar1-1 ndc80-K204E ipl1-2*	this study
KT3351	*MATα sds22-F177S*	this study
KT3353	*MATα ipl1-2 sds22-F177S*	this study
KT3355	*MATα glc7-Y136N*	this study
KT3358	*MATα ipl1-2 glc7-Y136N*	this study
KT3359	*MATα glc7-L74P*	this study
KT3361	*MATα ipl1-2 glc7-L74P*	this study
KT3363	*MATα glc7-Y92N R141K*	this study
KT3365	*MATα ipl1-2 glc7-Y92N R141K*	this study
KT3368	*MATα ipl1-2 ypi1-F74S*	this study
KT3370	*MATα ipl1-2 ypi1-F74L*	this study
KT3381	*MATα ipl1-2 sds22-W187R*	this study
KT3383	*MAT***a** *IPL1-13Myc:kanMX6*	this study

KT3385	*MATα duo1-S115F*	this study
KT3386	*MATα ipl1-2 duo1-S115F*	this study
KT3389	*MAT***a** *IPL1-13Myc:kanMX6 tco89-71*	this study
KT3391	*MAT***a** *IPL1-13Myc:kanMX6 glc7-L74P*	this study
KT3392	*MAT***a** *IPL1-13Myc:kanMX6 glc7-Y92N R141K*	this study
KT3395	*MAT***a** *IPL1-13Myc:kanMX6 shp1-105*	this study
KT3396	*MAT***a** *IPL1-13Myc:kanMX6 shp1-99*	this study
KT3400	*MAT***a** *ipl1-2 ybp2Δ::kanMX6*	this study
KT3401	*MATα ipl1-2 ybp2Δ::kanMX6*	this study
KT3403	*MATα ybp2Δ::kanMX6*	this study
KT3409	*MATα ipl1-2 duo1-S115F ybp2Δ::kanMX6*	this study
KT3410	*MATα duo1-S115F ybp2Δ::kanMX6*	this study
KT3412	*MATα shp1-99*	this study
KT3413	*MATα shp1-99 ipl1-2*	this study
KT3415	*MAT***a** *shp1-99 ipl1-2*	this study
KT3416	*MATα shp1-105*	this study
KT3417	*MAT***a** *shp1-105 ipl1-2*	this study
KT3419	*MATα shp1-105 ipl1-2*	this study
KT3424	*MAT***a** *GLC7-mCitrine:SpHis5 POM34-mCitrine:SpHis5 ipl1-2 shp1-105*	this study
KT3247	*MAT***a** *YPI1-13Myc:kanMX6 ipl1-2 shp1-105*	this study
KT3428	*MAT***a** *SDS22-mCitrine:SpHis5 POM34-mCherry:SpHis5 ipl1-2 shp1-105*	this study
KT3449	*MAT**a**/MATα ipl1-2/IPL1 duo1Δ::kanMX6 pRS306:duo1-S115F*	this study
KT3450	*MAT**a**/MATα ipl1-2/IPL1 ndc80Δ::kanMX6 pRS313:ndc80-K204E*	this study

[a] Stuart, J.S., Frederick, D.L., Varner, C.M., and Tatchell, K. (1994). The mutant type 1 protein phosphatase encoded by *glc7-1* from *Saccharomyces cerevisiae* fails to interact productively with the *GAC1*-encoded regulatory subunit. Mol Cell Biol *14*, 896-905.

[b] Frederick, D.L., and Tatchell, K. (1996). The *REG2* gene of *Saccharomyces cerevisiae* encodes a type1 protein phosphatase-binding protein that functions with Reg1p and the Snf1p protein kinase to regulate growth. Mol Cell Biol *16*, 2922-2931.

[c] Hsu, J.-Y., Sun, Z.-W., Li, X., Ruben, M., Tatchell, K., Bishop, D.K., Grushcow, J.M., Brame, C.J., Caldwell, J.A., Hunt, D.F., *et al.* (2000). Mitotic phosphorylation of histone H3 is goverened by Ipl1/aurora kinase and Glc7p/PP1 phosphatase in budding yeast and nematodes. Cell *102*, 279-291.

[d] Bharucha, J.P., Larson, J.R., Gao, L., Daves, L.K., and Tatchell, K. (2008). Ypi1, a Positive Regulator of Nuclear Protein Phosphatase Type 1 Activity in Saccharomyces cerevisiae. Mol Biol Cell *19*, 1032-1045.

[e] Tatchell, K., Makrantoni, V., Stark, M.J., and Robinson, L.C. (2011). Temperature-sensitive ipl1-2/Aurora B mutation is suppressed by mutations in TOR complex 1 via the Glc7/PP1 phosphatase. Proc Natl Acad Sci U S A *108*, 3994-3999.
