# Supplementary material for: Suppressors of ipl1-2 in Components of a Glc7 Phosphatase Complex, Cdc48 AAA ATPase, TORC1, and the Kinetochore
Source: G3 (Bethesda). 2012 Dec 1;2(12):1687–701. doi: 10.1534/g3.112.003814 (PMC3516489; doi:10.1534/g3.112.003814)
Supplement: Supporting Information [file supp_2.12.1687_TableS2.pdf]

Table S2 Primers Used

PRIMER	SEQUENCE, 5'- 3'
pBR-247F	GATGCAATTTCTATGCGCAC
pBR-559R	CAAGGAATGGTGCATGCAAG
DUO1-F	GATGAGAGGTTTTTGGACCTC
DUO1-R	CCTGTTGAAACTAAGGCATG
DUO1-Fa	CCTGCTCGTTGTACATATC
DUO1-Ra	CAACCTGTAGCCGTATTAAG
GLC7-F	CGTAACACAAGTTAACACC
GLC7-R	GAGTGATGATTGCATCTTCC
IPL1-F	GCAATTGCTCAAGTGAATG
IPL1-R	GCAATATACGATGCCGAAAAG
IPL1-SEQ	CGCTAAATCATCCGAATC
NDC80-F	GAATACATTCACAGGAGAGG
NDC80-R	GGTGGAATTGAACAAAATGC
NDC80-1644F	CTGTAGGTGGCTCTAATTG
NDC80-Fa	CTGGATCCCCAGATGGTTATAACTG
NDC80-Ra	CTTCTAGACCACCGTCTTCTTATTC
SDS22-F	CTCTCTCCTCCCTTTTTCTC
SDS22-R	GCGAACTTTAACTAGATGG
SHP1-F	GTGGACAGCTTAGTTAAGAC
SHP1-R	CTGCTCCAAAAGCAGTATTG

SHP1-303F	CAAGGTAGCAACGAGTACTTG
YBP2-F	CGTTTCGCTGTGAAACTAAC
YBP2-R	CGTACTTTCTGGGTTCCAATG
YBP2-571F	GTAACAACCAACGTTCAGG
YPI1-F	CCGTTTCTTAGCTTAAAGGC
YPI1-R	CAATACAGAATGCCAAGGG
IPL1-F2	GATACTAAGAAACAAGCCCTTTTGGGAAAATAAGCGGTTACGGATCCCCGGGTTAATTAA
IPL1-R1	ATTAATAGTGCCCTTCAAACGATTCTGTCATACTTTAATTGAATTCGAGCTCGTTTAAAC
NDC80-F2	GTTACGAAATTTGGAGTTTGAAACTGAACATAACGTAACAAATCGGATCCCCGGGTTAATTAA
NDC80-R1	CTTTGCTGTAGATTGCTCGGGTATTATATATCATTTATTTTAGAATTCGAGCTCGTTTAAAC
PDS1-F2	AGAAGGCCTCGATCCTGAAGAACTAGAGGACTTAGTTACTCGGATCCCCGGGTTAATTAA
PDS1-R1	ACGTGTATATATGTTGTGTGTATGTGAATGAGCAGTGGATGAATTCGAGCTCGTTTAAAC
SHP1-F2	CGCTGATCTGCTGAACTCCGTTGTCGTGCAAAGATGGGCACGGATCCCCGGGTTAATTAA
SHP1-R1	GTTGAAGTCTTTTCCCGTTTCTGTTTTTGTATATTTATGCGAATTCGAGCTCGTTTAAAC
